# How Can We Predict Accurate Electrochromic Shifts for Biochromophores? A Case Study on the Photosynthetic Reaction Center

**DOI:** 10.1021/acs.jctc.0c01152

**Published:** 2021-02-10

**Authors:** Abhishek Sirohiwal, Frank Neese, Dimitrios A. Pantazis

**Affiliations:** † Max-Planck-Institut für Kohlenforschung, Kaiser-Wilhelm-Platz 1, 45470 Mülheim an der Ruhr, Germany; ‡ Fakultät für Chemie und Biochemie, Ruhr-Universität Bochum, 44780 Bochum, Germany

## Abstract

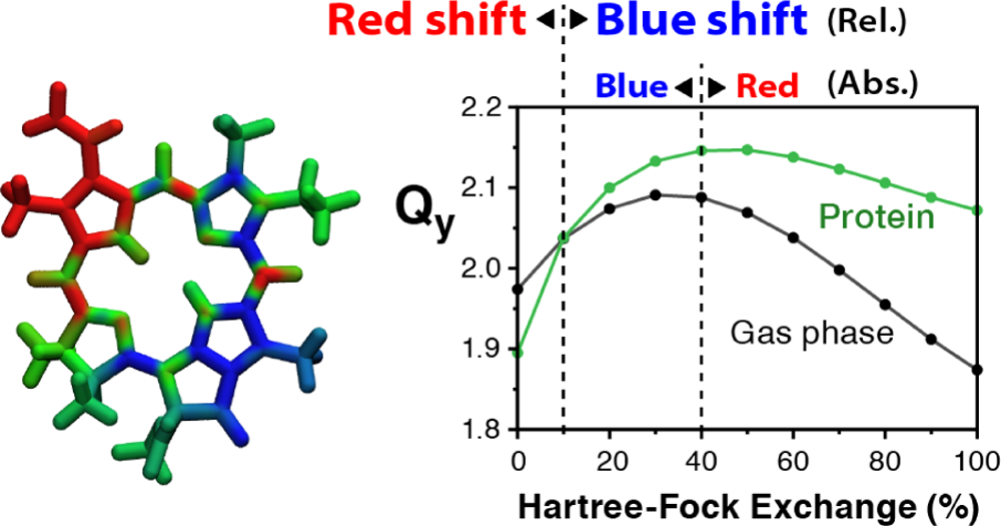

Protein-embedded chromophores are responsible for light harvesting, excitation energy transfer, and charge separation in photosynthesis. A critical part of the photosynthetic apparatus are reaction centers (RCs), which comprise groups of (bacterio)chlorophyll and (bacterio)pheophytin molecules that transform the excitation energy derived from light absorption into charge separation. The lowest excitation energies of individual pigments (site energies) are key for understanding photosynthetic systems, and form a prime target for quantum chemistry. A major theoretical challenge is to accurately describe the electrochromic (Stark) shifts in site energies produced by the inhomogeneous electric field of the protein matrix. Here, we present large-scale quantum mechanics/molecular mechanics calculations of electrochromic shifts for the RC chromophores of photosystem II (PSII) using various quantum chemical methods evaluated against the domain-based local pair natural orbital (DLPNO) implementation of the similarity-transformed equation of motion coupled cluster theory with single and double excitations (STEOM-CCSD). We show that certain range-separated density functionals (ωΒ97, ωΒ97X-V, ωΒ2PLYP, and LC-BLYP) correctly reproduce RC site energy shifts with time-dependent density functional theory (TD-DFT). The popular CAM-B3LYP functional underestimates the shifts and is not recommended. Global hybrid functionals are too insensitive to the environment and should be avoided, while nonhybrid functionals are strictly nonapplicable. Among the applicable approximate coupled cluster methods, the canonical versions of CC2 and ADC(2) were found to deviate significantly from the reference results both for the description of the lowest excited state and for the electrochromic shifts. By contrast, their spin-component-scaled (SCS) and particularly the scale-opposite-spin (SOS) variants compare well with the reference DLPNO-STEOM-CCSD and the best range-separated DFT methods. The emergence of RC excitation asymmetry is discussed in terms of intrinsic and protein electrostatic potentials. In addition, we evaluate a minimal structural scaffold of PSII, the D1–D2–Cyt_B559_ RC complex often employed in experimental studies, and show that it would have the same site energy distribution of RC chromophores as the full PSII supercomplex, but only under the unlikely conditions that the core protein organization and cofactor arrangement remain identical to those of the intact enzyme.

## 1 Introduction

The input of energy into the biosphere is mediated by the conversion of sunlight to chemical energy in the form of separated charges, which drive the redox transformations of photosynthesis.^1−3^ This process occurs in protein-embedded assemblies of (bacterio)chlorophylls and (bacterio)pheophytins, the reaction centers (RCs) of biological photosystems. There are different types of RCs in biology. They have extensive similarities, for example, in their two-branch arrangement of chromophores, but also specific differences, for example, in the nature of terminal electron donors and acceptors, in the chemical nature of constituent pigments, and the functional asymmetry of the branches.

**Figure 1:**
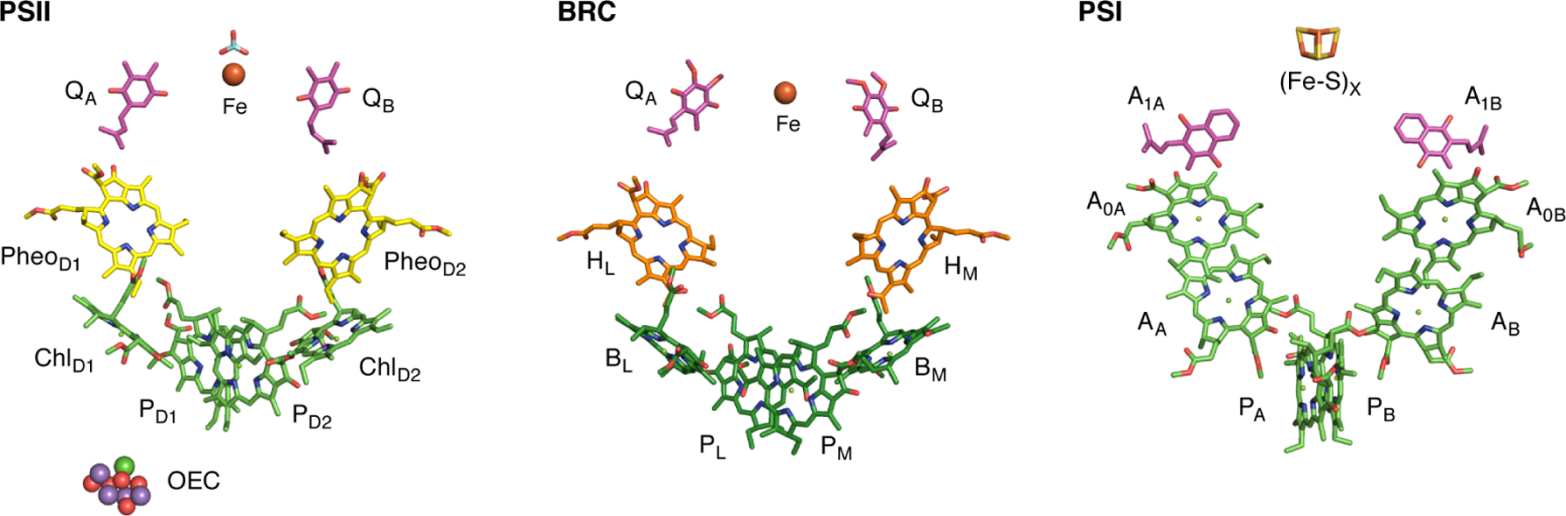
RC chromophores of photosystem II (left), the BRC (middle), and photosystem I (right).

Figure 1 depicts the major components that comprise the RCs of photosystem II (PSII), the bacterial RC (BRC), and photosystem I (PSI). The RC of the water-oxidizing PSII consists of chlorophyll *a* and pheophytin *a* molecules arranged in a pseudosymmetric fashion along two branches known as the D1 and D2 branches, from the conventional designation of the proteins that accommodate them. The D1 branch is active in electron transfer from the charge separation site, whereas the D2 branch is presumably involved in photoprotection. In PSII, the terminal electron donor is water, while the terminal acceptor is the mobile electron carrier plastoquinone Q_B_. Water is oxidized at the site of the oxygen-evolving complex (OEC),^4−9^ which is electronically coupled to the RC chlorophylls via one of the two redox-active tyrosine^10^ residues of PSII. The RC of BRC is composed of the far-red absorbing bacteriochlorophyll *a* and bacteriopheophytin *a*, arranged symmetrically along the L and M branches.^11^ Despite their differences, the BRC resembles PSII in using only the L-branch as functionally active. Finally, the RC in photosystem I is made up of two quasisymmetric branches.^12^ In analogy to PSII, PSI contains a central pair of coupled chlorophyll *a* and modified chlorophyll *a* (Chl *a*′) pigments, whereas the rest of the chromophores are chlorophyll *a* molecules. However, unlike PSII and the BRC, electron transfer in PSI is active along both branches, even though branch A may be favored.^13^

A common feature of RCs is that despite the apparent structural symmetry of the pigments themselves, there is functional asymmetry in excitation, charge separation, and electron transfer. The cases of the PSII and the BRC are of prime importance because of exclusive unidirectional electron transfer through a dominant branch. The excitonic structure of the pigments in BRC is well understood because of the resolved absorption features, and it is known that the special pair (P_L_/P_M_) forms the sink of excitation energy and initiates electron transfer.^14,15^ However, our understanding of the RC in PSII is incomplete. Uncertainties remain about the site energies of individual pigments, the nature and localization of initial excitation, the identity of the primary electron donor, the possible charge transfer states that can be created upon excitation, and the subsequent charge separation pathways.^16−27^

Accurate calculation of site energies is a key requirement for understanding the various processes taking place within the RC, because the site energies of the chromophores determine the trapping site of excitation energy, as well as the nature and directionality of charge separation. The fundamental understanding of these systems can be aided by explicit quantum chemical calculations of their electronic structure. Challenges for the quantum chemical description of biochromophores include the requirement for atomistic modeling of protein–pigment interactions and the requirement for accurate and reliable methods to compute excited states.^28−33^ Quantum mechanics/molecular mechanics (QM/MM) approaches^34,35^ can account explicitly for protein–pigment interactions and include the steric and electrostatic effects of the protein: the chromophore of interest and a few key components of its immediate environment are treated using a QM method and the rest of the protein using an MM model. QM/MM approaches are required to understand the role of the protein environment in modulating the properties of individual chromophores, differentiating them excitonically, and creating the asymmetries that underpin the function of RCs.^25^ The protein matrix fine-tunes the local properties of individual chromophores by either blue-shifting or red-shifting their “intrinsic”, often undifferentiated site energies, under the influence of a structured, anisotropic electrostatic environment. This can be viewed as a protein-induced Stark effect,^36,37^ and the corresponding electrochromic shifts become key elements in deciphering the functional role of the protein.

Assuming that the geometries of the chromophores are optimized within a QM/MM approach using an appropriate QM method and that electrostatic effects of the protein matrix are explicitly considered in excited state calculations, the focus is placed on the method used for computing these excited states. Here, the paramount requirement is the correct response of computed excitations to protein matrix electrostatics, that is, the electrochromic shifts. In the simplest sense, this can be stated in terms of the response of the computed site energies of individual RC chromophores (the “Stark shift” of the first excited state transition energy) to the anisotropic electric field of the environment.

The goal of the present work is to understand how different QM methods perform for the calculation of electrochromic shifts in the context of a QM/MM approach for the chlorophyll and pheophytin site energies in the RC of PSII. Our reference method is the recently developed domain-based local pair natural orbital (DLPNO) implementation of the similarity-transformed equation of motion coupled cluster theory with single and double excitations (STEOM-CCSD).^38−44^ Against this method, we test a variety of commonly used density functionals within the time-dependent density functional theory approach (TD-DFT), semiempirical approaches (ZINDO/S),^45^ and more approximate wavefunction-based methods such as CC2 (approximate coupled cluster with singles and doubles)^46^ and ADC(2)^47,48^ (algebraic diagrammatic construction through second order), along with their spin-component-scaled (SCS) and scaled-opposite-spin (SOS)^49−51^ variants. The results provide a clear hierarchy of methods for the calculation of electrochromic shifts and we expect that the methodological conclusions are transferable to any similar problem. Furthermore, the results provide important physical insights into PSII itself, describing the emergence of excitonic asymmetry within the RC, quantifying the differentiation of site energies between the active and inactive branches, and rationalizing the origin of red- and blue-shifting for crucial RC chromophores. Finally, the results obtained with selective omission of light-harvesting antennae and ancillary proteins suggest that the electrochromic shifts on RC chromophores originate primarily from core proteins D1–D2–Cyt_b559_, provided these are in their native conformation, and that Chl_D1_ is the pigment most affected by the electrostatic effect of the intrinsic antennae (CP43 and CP47) and the extrinsic proteins of PSII.

## 2 Methodology

### 2.1 Classical Molecular Dynamics

**Figure 2:**
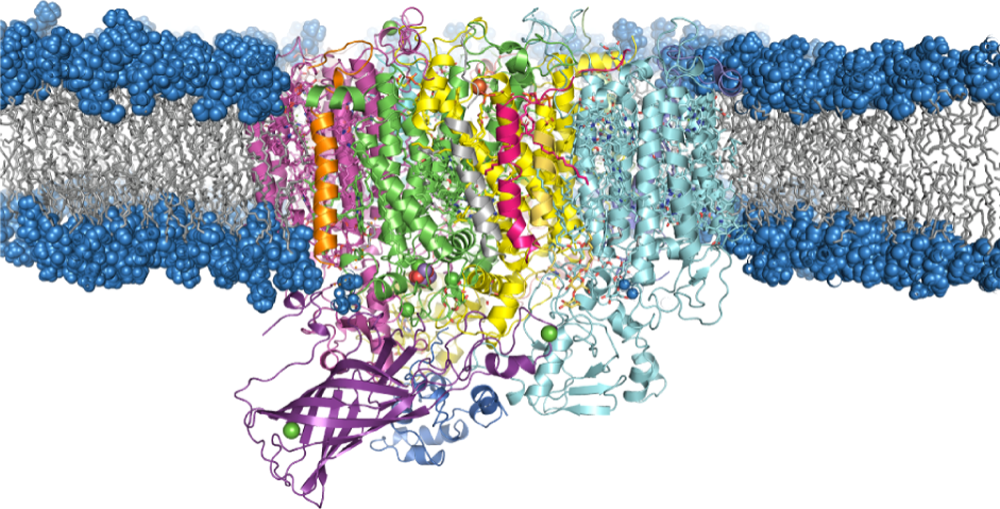
Cutaway side view of the all-atom model of the PSII monomer used in the present study, embedded in the POPC lipid bilayer. Water and salt ions of the simulation box are omitted for clarity.

The 1.9 Å resolution crystal structure of Photosystem II (3WU2)^52^ was used to build the entire MM model. The PSII monomer was embedded^53^ inside a lipid bilayer and water molecules were added in the stromal and lumenal sides (i.e., along the *z*-axis, normal to the membrane plane). Na^+^ and Cl^–^ counterions were added to reach a physiological salt concentration of 0.15 M. The final dimensions of the complete system were 176 Å × 176 Å × 160 Å, consisting of 512,341 atoms in total (Figure 2). Electrostatic charges for all cofactors were derived using the Merz–Kollman restrained electrostatic potential (MK-RESP) strategy.^54^ Parameters for protein residues, organic cofactors, and water and lipid bilayers were derived from the Amber14SB,^55^ GAFF2,^56^ TIP3P,^57^ and LIPID17 force-fields,^58,59^ respectively.

The complete system was relaxed using systematic minimization. The system was heated from 10 to 100 K in a succession of 5 ps in the *NVT* ensemble. Following that, the temperature was slowly increased from 100 to 303 K in the *NPT* ensemble for 125 ps, along with a positional restraint (20 kcal mol^–1^ Å^–2^) on the C_α_ atoms of amino acids. The system was further equilibrated in the *NPT* ensemble for 2 ns, maintaining a 20 kcal mol^–1^ Å^–2^ restraint on C_α_ atoms, at 303 K and 1 atm pressure. The temperature was controlled using Langevin dynamics^60^ with a collision frequency of 5 ps^–1^ and the pressure was controlled using the Berendsen barostat^61^ with anisotropic pressure scaling with a relaxation time of 2 ps. The SHAKE algorithm^62^ was used to constrain bonds involving hydrogen atoms, which allowed for a time step of 2 fs. Particle mesh Ewald^63^ method was used to treat electrostatic interactions with a 10 Å cutoff. All these calculations were performed using the AMBER18 molecular dynamics package.^64,65^

### 2.2 QM/MM Calculations

A protein structural configuration was obtained after clustering the frames^66−68^ obtained from the classical propagation. To set up the system for the QM/MM computations, we chose the entire PSII monomer and all waters (including internal cavity waters) at a distance of nearly 7 Å around the protein. The entire system for the QM/MM contains a total of 76,056 atoms. Our QM/MM approach is based on the electrostatic embedding technique. The QM/MM optimization was performed using the ChemShell 3.7 code.^69−71^ The in-built DL-POLY module^72^ handled the MM region, whereas the ORCA package^73^ was used to treat the QM region. Each participating chromophore of the RC was optimized individually, except the special pair (P_D1_/P_D2_), which was optimized as a single unit. Such a procedure correctly describes the polarization effects due to their close proximity and reproduces the vinyl symmetry break, which is otherwise poorly predicted using the MM force field. Besides chromophores, the axial ligands were also considered in the QM region; phytyl chains were truncated and only treated in the MM region. The hydrogen link atom approach was employed to cut through covalent bonds, and the charge-shift method was used to avoid overpolarization of the QM region.

During geometry optimizations, the complete system is divided into two parts, that is, active and static. The active region consists of atoms within the QM and MM regions, which are free to move during the geometry optimization iterations, whereas the atoms in the static region remain fixed throughout and only act as part of the electrostatic environment. In the case of the individually optimized chromophores (i.e., Chl_D1_, Chl_D2_, Pheo_D1_, and Pheo_D2_), all protein components within 13 Å (ca. 1300–1600 atoms) around the QM region were considered in the active region, whereas a larger active region (∼15 Å, ca. 2650 atoms) was chosen around the special pair (P_D1_/P_D2_). The Perdew–Burke–Ernzerhof (PBE) functional^74^ was used to optimize the QM regions using the Def2-TZVP basis set,^75^ along with the D3(BJ) dispersion corrections.^76,77^ A higher DFT integration grid (grid6 in ORCA convention) was used in all calculations. The resolution of identity approximation^78,79^ was used to speed up the calculation of Coulomb integrals with a matching auxiliary basis set.^80^ The QM/MM geometry optimization convergence criterion for the maximum gradient component was set to 4.5 × 10^–4^ hartree/bohr. The criterion for the maximum step components was 1.8 × 10^–3^ bohr, in the case of the root mean square (rms) gradient 3.0 × 10^–4^ hartree/bohr and for the rms step, the tolerance was 1.2 × 10^–3^ bohr. The convergence criterion for energy change was set to 1.0 × 10^–6^ hartree.

The direct use of the crystallographic coordinates of biochromophores is inappropriate for quantum chemical studies^81^ because crystallographic structures lack critical precise information on bond-length alternation. Because of the nature of the participating Frontier orbitals (π → π*) in the low-energy excitations, their energetics are sensitively dependent on the ground state structure and conjugation. The geometries must, therefore, be optimized at an appropriate QM level before investigating excited state properties or other sensitive aspects of the electronic structure such as spin density distributions. This point has been raised in several studies of biochromophores over the years.^32,33,81−85^ In a recent example, Kaila and coworkers^86^ have specifically advocated the use of optimized structures of RC chromophores, having observed shifts up to 0.3 eV (in Q_*y*_) between the structure derived from the crystal and quantum chemically optimized geometries. Unfortunately, the direct use of crystallographic coordinates can still be encountered in theoretical studies of biochromophores, contributing to confusion through unreliable computational results.

### 2.3 Calculations of Excitation Energies

Vertical excitation energies on the optimized QM/MM geometries were computed using wave function-based methods and TD-DFT. The electrostatic effect of the protein environment on the QM ground and excited states was included through the MK-RESP point charges, which were applied for the whole PSII monomer without cutoffs. The recently proposed^38,41,42^ combination of the ground state DLPNO method^43,87^ with the STEOM approach allows the decoupling of single excitations from the doubles, thus reducing the final diagonalization step to the size of the space of single excitations similar to TD-DFT. Unlike TD-DFT, the effect of the doubles is retained and the additional implicit triples correction significantly improves the description of charge transfer states. The resulting DLPNO-STEOM-CCSD method thus does not rely on the less robust perturbative approximation to EOM-CCSD as the more approximate CC2 and ADC(2) approaches. In the DLPNO-STEOM-CCSD calculations reported in this work, we computed six excited states for each RC chromophore, using the Def2-TZVP(-f) basis-set. The RIJCOSX approximation^88,89^ was used to speed up the calculations throughout. “TightPNO” settings were applied for all DLPNO calculations. The *T*_CutPNOsingles_ cutoff was set to 6.6 × 10^–10^ and the active space selection keywords “Othresh” and “Vthresh” were set to 5.0 × 10^–3^. All DLPNO-STEOM-CCSD calculations were performed with a development version of ORCA 4.2.^73^

The vertical excitation energies for all chromophores were also computed with the CC2^46,90,91^ and ADC(2)^47,48^ methods using the resolution-of-identity approximation^90^ with the Def2-TZVP(-f) basis sets and corresponding auxiliary basis sets.^92^ The frozen core approximation is used throughout, where 1s orbitals of all nonhydrogen atoms are not included in the correlation treatment. In addition, SCS (*C*_same-spin_ = 0.33, *C*_opposite-spin_ = 1.20) and SOS^49,93^ (*C*_same-spin_ = 0, *C*_opposite-spin_ = 1.3) variants of the CC2 and ADC(2) methods were evaluated. All CC2 and ADC(2) calculations were performed using Turbomole 7.5.^94,95^

All TD-DFT calculations were performed with ORCA 4.2, using a variety of functionals. These can be classified into the generalized gradient approximation (GGA) functionals BP86,^96,97^ BLYP^96,98^ and PBE,^74^ global hybrids B3LYP^99^ (20% exact exchange, or Hartree–Fock exchange, HFX), PBE0^100^ (25% HFX), B1LYP^101^ (25% HFX), BHandHLYP^102^ (50% HFX), and global double hybrid B2PLYP^103^ (50% HFX with the perturbative second-order correlation part). Range-separated hybrid functionals containing variable HFX at short and long range are also considered: ωB97^104^ (HFX = 0–100%, ω = 0.4), ωB97X-V^105^ (HFX = 16.7–100%, ω = 0.30), CAM-B3LYP^106^ (HFX = 19–65%, μ = 0.33) and LC-BLYP^107^ (HFX = 0–100%, ω = 0.33), and range-separated double hybrid ωB2PLYP^108^ (HFX = 53–100%, ω = 0.30). Additionally, a modified version of CAM-B3LYP was considered, with the attenuation parameter μ set to 0.14, based on a recommendation by Saito et al.^109^ This modified version is denoted as CAM-B3LYP* in the present work. All TD-DFT computations were performed using the Def2-TZVP basis sets^75^ and the corresponding auxiliary basis sets^80^ with the RI-J and COSX approximations.^88,89^ The first 10 excited states were computed for each RC chromophore. Very tight SCF convergence criteria were applied throughout, along with higher integration grids (Grid6, GridX7). In addition, excited state calculations were performed using the semiempirical ZINDO/S method.^45^

## 3 Results and Discussion

### 3.1 Site Energies in the Gas Phase

**Scheme 1:**
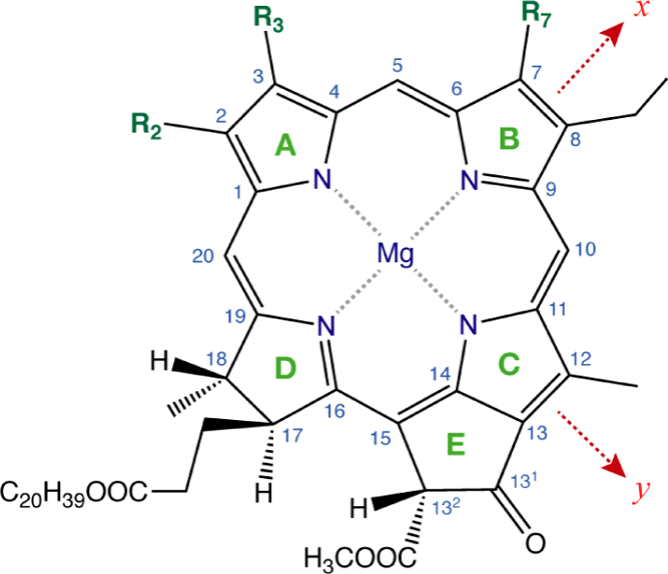
Chlorin Numbering Scheme with Indication of Conventionally Defined *x* and *y* Axes For chlorophyll *a*, the R substituents are R_2_ = CH_3_, R_3_ = C_2_H_3_, and R_7_ = CH_3_. In pheophytin *a*, the Mg ion is replaced by two protons on the N atoms of rings A and C.

The origin and nature of the low-energy excited states in (bacterio)chlorophylls are typically conceptualized in the framework of the Gouterman model,^110,111^ which attempts to describe the lowest energy absorption feature (the Q-band) and the higher-energy feature (the B-band) in terms of four frontier orbitals, HOMO – 1, highest occupied molecular orbital (HOMO), lowest unoccupied molecular orbital (LUMO), and LUMO + 1. The lowest energy excitation (S_0_ → S_1_) is commonly referred to as Q_*y*_, where *y* denotes the directionality of the transition dipole moment in the macrocyclic plane. In this case, the majority of the contribution are derived from the HOMO → LUMO transition and secondarily from the HOMO – 1 → LUMO + 1 transition, both *y*-polarized in the idealized porphyrin parent system as opposed to the *x*-polarized HOMO → LUMO + 1 and HOMO – 1 → LUMO transitions. The definition of directions is shown in Scheme 1, which indicates the standard nomenclature and labeling of the atoms and the five-membered rings of the chlorin.

**Figure 3:**
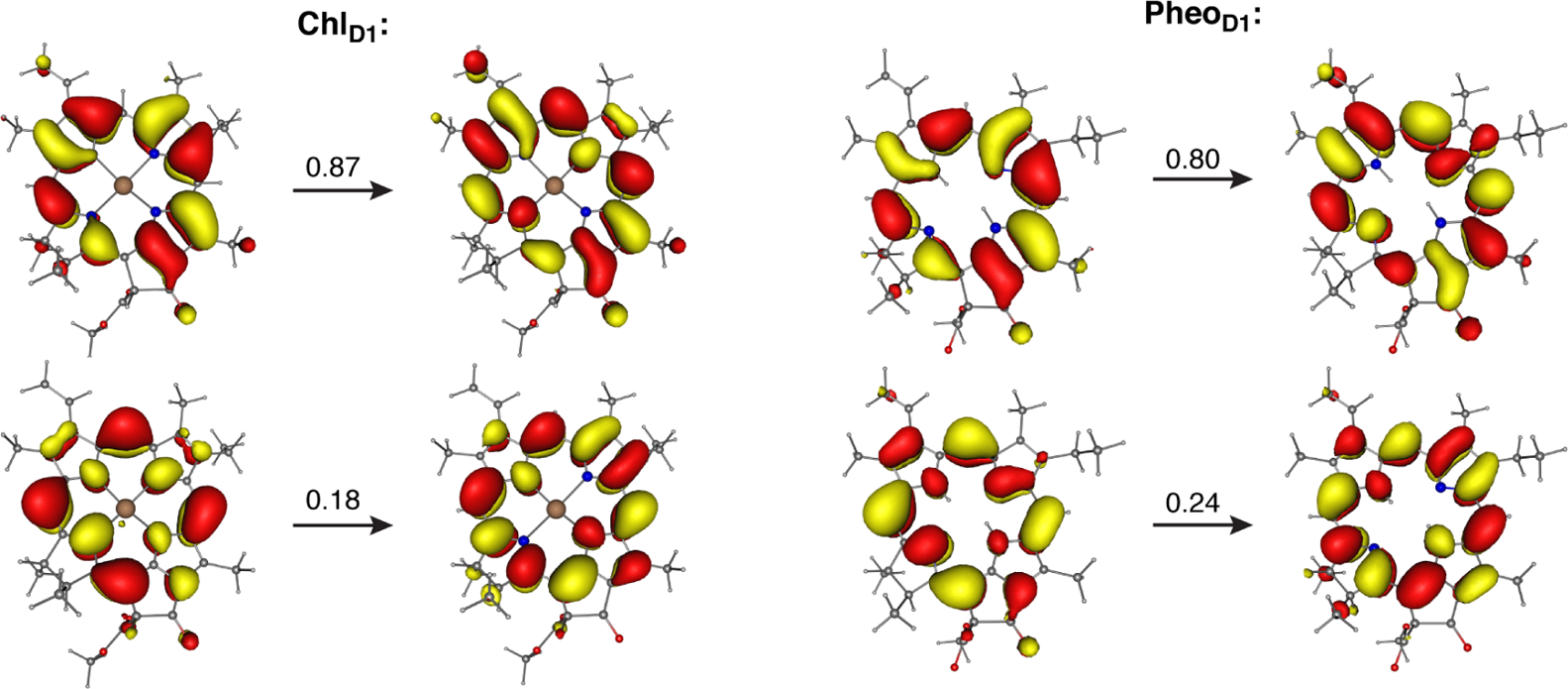
NTOs describing the Q_*y*_ transition (first excited state, S_1_), for the Chl_D1_ and Pheo_D1_ chromophores of the PSII RC. The corresponding weights of excitations are shown above the arrows from TD-DFT calculations with the ωΒ97X-V functional.

Using QM/MM optimized geometries of the individual RC chromophores of PSII, we first computed vertical excitation energies in the gas phase, that is, in the absence of point charges. The excitation energies for all chromophores are listed in Table 1. Figure 3 shows the description of the lowest energy excitation in terms of natural transition orbitals (NTOs) for one of the chlorophylls (Chl_D1_) and one of the pheophytins (Pheo_D1_) of the RC, as obtained with the ωB97X-V density functional. The dominant transition in both cases corresponds to the HOMO → LUMO excitation and the nature of the NTOs is very similar for the chlorophyll and pheophytin molecules because the Mg ion of the chlorophyll does not contribute to the chlorin Frontier orbitals involved in this excitation.

**Table 1:** Absolute Values in eV of the Q_*y*_ Excitation Energy in Gas-phase Calculations of PSII RC Chromophores Using Various Quantum Chemical Methods^a^

method	P_D1_	P_D2_	Chl_D1_	Chl_D2_	Pheo_D1_	Pheo_D2_
DLPNO-STEOM-CCSD	1.633 (0.22)	1.635 (0.20)	1.642 (0.23)	1.649 (0.23)	1.601 (0.15)	1.591 (0.16)
CC2	2.126 (0.19)	2.131 (0.16)	2.128 (0.21)	2.117 (0.21)	2.089 (0.17)	2.084 (0.17)
ADC(2)	1.887 (0.21)	1.898 (0.18)	1.896 (0.24)	1.883 (0.25)	1.873 (0.23)	1.863 (0.23)
SCS-CC2	2.064 (0.23)	2.071 (0.20)	2.068 (0.24)	2.071 (0.24)	2.023 (0.17)	2.023 (0.17)
SCS-ADC(2)	1.946 (0.26)	1.952 (0.23)	1.952 (0.28)	1.955 (0.27)	1.930 (0.21)	1.929 (0.21)
SOS-CC2	2.022 (0.21)	2.027 (0.19)	2.027 (0.23)	2.037 (0.22)	2.002 (0.17)	2.004 (0.17)
SOS-ADC(2)	1.936 (0.24)	1.940 (0.21)	1.942 (0.26)	1.952 (0.24)	1.937 (0.19)	1.939 (0.19)
ωΒ97	1.858 (0.22)	1.857 (0.20)	1.853 (0.23)	1.881 (0.22)	1.860 (0.18)	1.868 (0.18)
ωΒ97X-V	1.925 (0.23)	1.927 (0.21)	1.920 (0.24)	1.943 (0.23)	1.923 (0.19)	1.930 (0.19)
ωB2PLYP	1.895 (0.25)	1.897 (0.23)	1.893 (0.26)	1.909 (0.25)	1.873 (0.20)	1.878 (0.20)
LC-BLYP	1.890 (0.21)	1.889 (0.19)	1.886 (0.22)	1.907 (0.22)	1.881 (0.17)	1.887 (0.17)
CAM-B3LYP	2.012 (0.25)	2.014 (0.22)	2.007 (0.26)	2.022 (0.25)	2.010 (0.20)	2.013 (0.20)
CAM-B3LYP*	2.055 (0.24)	2.058 (0.22)	2.050 (0.26)	2.060 (0.25)	2.060 (0.20)	2.061 (0.20)
B2PLYP	2.032 (0.28)	2.041 (0.25)	2.032 (0.30)	2.033 (0.29)	1.991 (0.23)	1.989 (0.23)
BHandHLYP	2.059 (0.28)	2.062 (0.25)	2.053 (0.30)	2.068 (0.29)	2.070 (0.23)	2.072 (0.24)
B1LYP	2.076 (0.25)	2.081 (0.22)	2.071 (0.27)	2.080 (0.26)	2.085 (0.20)	2.086 (0.21)
B3LYP	2.066 (0.24)	2.072 (0.21)	2.062 (0.26)	2.070 (0.25)	2.075 (0.19)	2.076 (0.20)
PBE0	2.091 (0.25)	2.097 (0.22)	2.086 (0.27)	2.096 (0.26)	2.100 (0.21)	2.101 (0.21)
PBE	1.977^b^ (0.17)	1.990^b^ (0.11)	1.971 (0.20)	1.979^b^ (0.19)	1.985 (0.14)	1.984 (0.15)
BLYP	1.965^b^ (0.16)	1.977^b^ (0.10)	1.959 (0.19)	1.967^b^ (0.19)	1.974 (0.14)	1.973 (0.14)
BP86	1.976^b^ (0.16)	1.989^b^ (0.10)	1.969 (0.20)	1.977^b^ (0.19)	1.985 (0.14)	1.984 (0.15)
ZINDO/S	1.596 (0.34)	1.607 (0.32)	1.593 (0.35)	1.608 (0.34)	1.615 (0.24)	1.616 (0.24)

a The oscillator strengths are shown in parentheses.

b The energies of P_D1_, P_D2_, and Chl_D2_ reported for PBE, BLYP, and BP86 correspond to the second excited state (S_2_), because the nature of the S_1_ state is incorrect with these functionals.

Nearly all methods used in the present work describe the electronic nature of the lowest energy excited state correctly and produce very similar results. However, there are two notable and important exceptions. First, the low-energy spectrum computed with the GGA functionals (PBE, BP86, and BLYP) contains “ghost states” in the case of P_D1_, P_D2_, and Chl_D2_, and it is the second (S_2_) rather than the first excited state that can be associated with “Q_*y*_” for these functionals. Similar concerns regarding ghost states were raised by List et al.^112^ in a study involving the bacteriochlorophyll *a* pigment in the Fenna–Matthew–Olson (FMO) complex. Therefore, GGA functionals should better be avoided altogether. Second, both CC2 and ADC(2) predict a mixed character of the first excited state for the RC chlorophylls: the HOMO – 1 → LUMO excitation has a significant contribution in the S_1_ state (26–37%) and the HOMO → LUMO and HOMO – 1 → LUMO + 1 excitations have much smaller contribution than anticipated (see Supporting Information). The SOS and SCS variants of the CC2 and ADC(2) correct this failure for all chromophores, showing the HOMO → LUMO and HOMO – 1 → LUMO + 1 transitions to be the dominant contributors to S_1_ (Q_*y*_), as expected. In this context, it is worth mentioning that past studies have also suggested that spin-scaled variants of CC2 and ADC(2) provide improved overall performance over their unscaled counterparts particularly for π → π* excitations.^49,113,114^

Based on the present results, even before discussing energies and electrochromic shifts, it appears that the spin-scaled variants are to be preferred over the canonical forms of CC2 and ADC(2) simply on the basis of electronic structure alone. It is important to stress that deficiencies in any given method are not revealed, and cannot be deduced, by mere inspection of excitation energies. Instead, the nature of the transition needs to be studied in detail. This remark pertains also to the evaluation of past literature reports, when only energies are mentioned without further analysis of the electronic structure. With the exception of GGA functionals and the CC2 and ADC(2) methods, all other methods considered in this work yield the correct first excited state.

The gas-phase results show that although different methods provide numerically different results in an absolute sense, the computed energies using any given method are very similar for all RC chromophores. Analogous results have been reported before when calculations are performed in the absence of the protein.^25,86,115,116^ Importantly, even when the geometric strain imposed by the protein is included implicitly by using QM/MM-optimized geometries for these gas-phase excited state calculations, as in the present work, no excitonic asymmetry in terms of strongly differentiated site energies arises in the RC.^25^ This starkly highlights the necessity of adequately capturing the effect of protein electrostatics, because this is the only way of achieving a meaningful computational representation of the physical system.

### 3.2 Effect of the Protein Matrix: Electrochromic Shifts

The electrostatic effects of the protein matrix modulate the excited state properties of biochromophores.^25,28,31,117^ For the purposes of this work, we define the “electrochromic shift” of the chromophores as the difference in the lowest excitation energy between the isolated chromophore in vacuo and the same chromophore embedded in the protein point charge field. In our approach, the same QM/MM optimized geometry is used for both calculations, therefore structural effects are already included and do not appear as additional terms. The electrochromic shift as defined here is a purely computational measure of the ability of a given method to respond to the environment, which in our approach is represented by point charges.

As a first step, it is important to clarify the validity of using DLPNO-STEOM-CCSD as a reference method. An experimental equivalent of electrochromic shifts according to the above definition is not available, but we can deduce a semiquantitative measure of such a shift by using the experimental gas-phase absorption spectrum^118,119^ of chlorophyll *a*. On the other hand, we do not have any experimental site energy for any specific chlorophyll under consideration: the absorption spectra of light-harvesting and RC complexes are convoluted, hindering the definitive assignment of individual site energies. Nevertheless, we can compare the gas-phase experimental absorption maximum^118,119^ with the known absorption spectrum of PSII core complexes (PSII-CC) or RC complexes (PSII-RCC, which contain only proteins D1, D2, and cytochrome Cyt_b559_), by assigning the absorption maximum of PSII samples to the lowest excitonic state of Chl_D1_. This approximation is inexact but justifiable because this is the pigment with the lowest site energy, according to the results obtained in this study and according to previous observations.^16,18,26,120−125^ The shift in this case is estimated as ca. 0.12 eV. We note that this is an order of magnitude larger than the magnitude of vibronic effects determined for Chl *a*.^83^ The computational comparison can be done between a gas-phase optimized Chl *a* molecule and the QM/MM optimized and protein-embedded Chl_D1_. Corresponding values of these shifts are computed using DLPNO-STEOM-CCSD and other candidate wavefunction-based reference methods, that is canonical CC2 and ADC(2) and their respective variants (see Table 2). It is clear that the canonical forms of the CC2 and ADC(2) underestimate the magnitude of the shift, whereas their respective spin-scaled variants perform better. However, we find that DLPNO-STEOM-CCSD gives the best agreement with the “quasi-experimental” shift, with a predicted value of 0.092 eV. The only other method that comes close is SOS-CC2 with a computed shift of 0.061 eV. Therefore, DLPNO-STEOM-CCSD is confirmed as the preferred reference method to benchmark more approximate approaches.

**Table 2:** Comparison of the Electrochromic Shift (in eV) Defined as the First Excited State Energy of Chl_D1_ in the Protein (QM/MM Treatment in the case of Computed Values) Minus the First Excited State Energy of Gas-phase Chl *a*, as Deduced from Experiment and Computed by Selected Post-Hartree–Fock Methods

	gas-phase	PSII matrix (Chl_D1_)	shift
experiment	∼1.94	∼1.82^a^	∼−0.12
DLPNO-STEOM-CCSD	1.667	1.575	–0.092
CC2	2.146	2.098	–0.048
ADC(2)	1.925	1.878	–0.047
SCS-CC2	2.064	2.011	–0.053
SCS-ADC(2)	1.947	1.898	–0.049
SOS-CC2	2.021	1.961	–0.061
SOS-ADC(2)	1.936	1.879	–0.057

a The absorption maximum depends on the organism and sample preparation, therefore we have used the average literature value of 1.82 eV (680 nm). The gas-phase and PSII matrix-based structural optimizations were performed using the PBE functional with the def2-TZVP basis set, in line with previous work^83^ on gas-phase Chl *a*.

**Figure 4:**
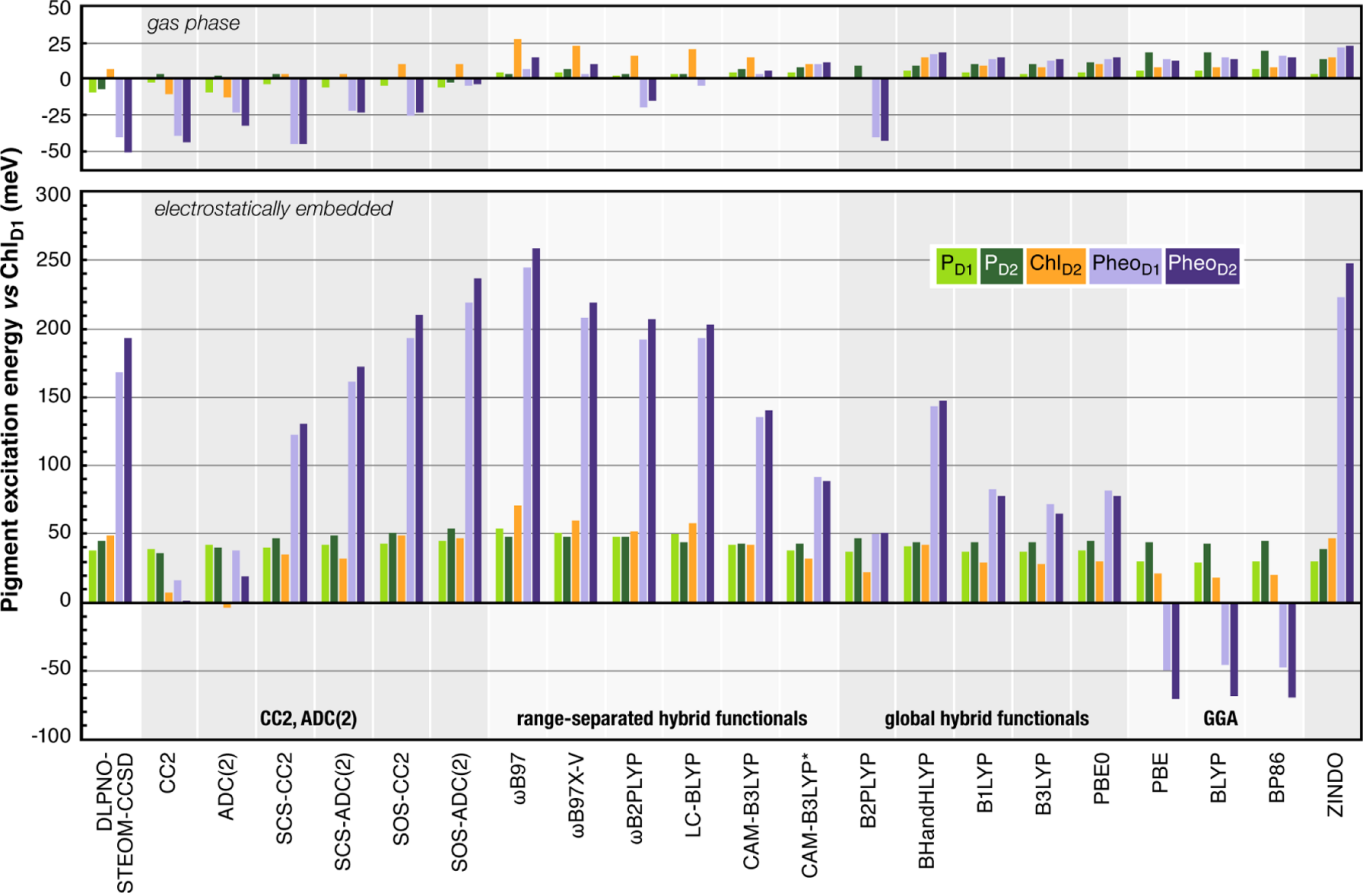
Relative Q_*y*_ excitation energies of the RC pigments obtained with various methods studied in this work, referenced to the Chl_D1_ first excitation energy. Comparison of the gas-phase results (top panel) with electrostatically embedded results (bottom panel).

**Figure 5:**
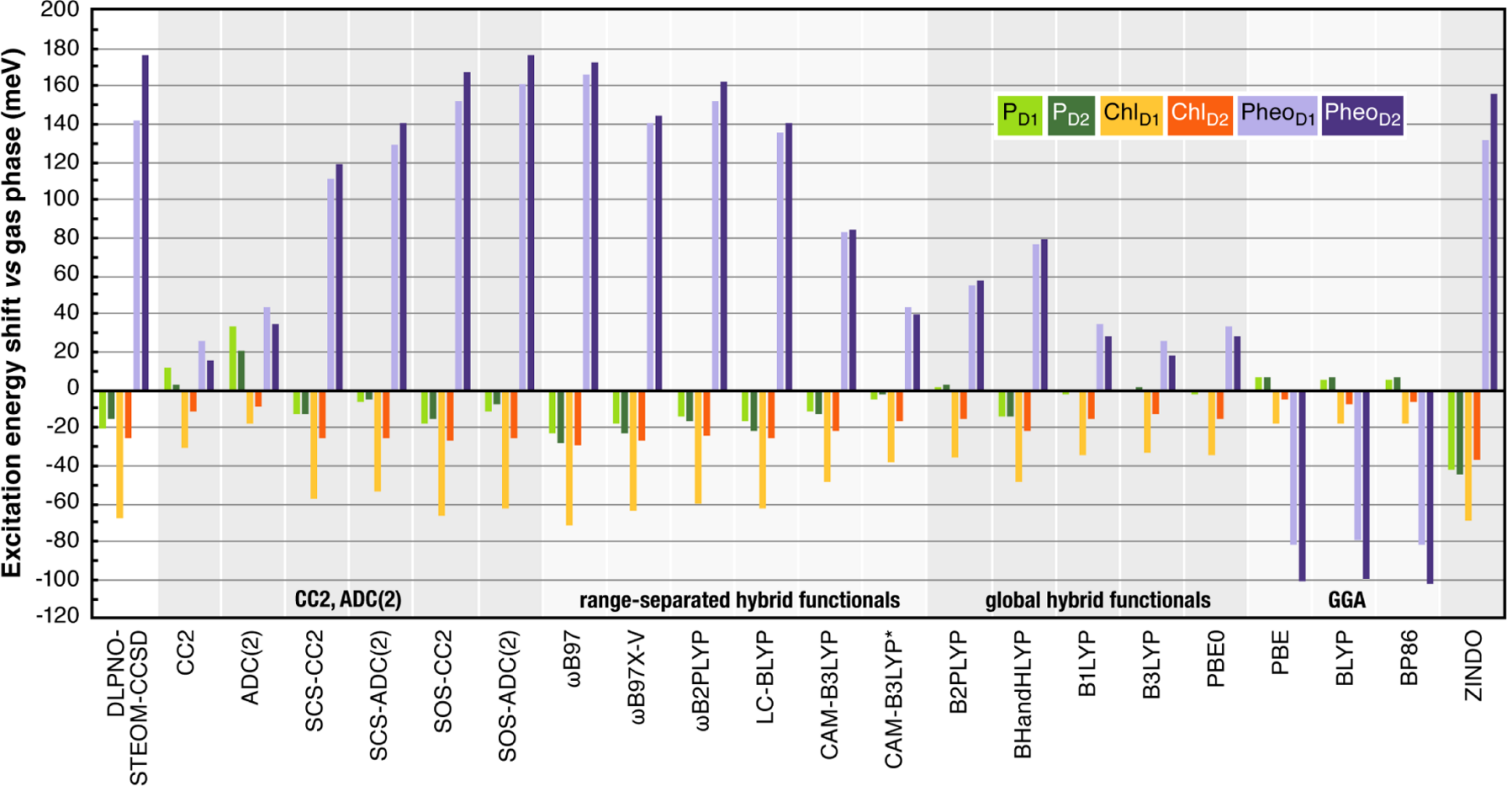
Absolute shifts in the Q_*y*_ excitation energies (in meV) of RC pigments upon electrostatic embedding, obtained with different methods.

In the following, we report the results of excited state calculations within the complete PSII matrix, with the electrostatic environment represented as point charges. Table 3 lists the absolute site energies obtained using different methods and Figure 4 shows a graphical comparison of relative excitation energies for gas-phase and electrostatically embedded calculations, referenced to the Chl_D1_ excitation energy. Table 4 lists the electrochromic shifts, which are also graphically depicted in the histogram of Figure 5.

**Table 3:** Absolute Value of the Q_*y*_ Excitation Energy (with the Electrostatic Embedding Using the QM/MM Optimized Structure) Using Various Quantum Chemical Methods^a^

method	P_D1_	P_D2_	Chl_D1_	Chl_D2_	Pheo_D1_	Pheo_D2_
DLPNO-STEOM-CCSD	1.613 (0.27)	1.620 (0.26)	1.575 (0.30)	1.624 (0.26)	1.743 (0.13)	1.768 (0.12)
CC2	2.137 (0.25)	2.134 (0.20)	2.098 (0.27)	2.105 (0.25)	2.114 (0.19)	2.099 (0.18)
ADC (2)	1.920 (0.28)	1.918 (0.25)	1.878 (0.29)	1.874 (0.29)	1.916 (0.23)	1.897 (0.22)
SCS-CC2	2.051(0.27)	2.058 (0.24)	2.011 (0.30)	2.046 (0.27)	2.134 (0.17)	2.142 (0.17)
SCS-ADC(2)	1.940 (0.31)	1.947 (0.29)	1.898 (0.35)	1.930 (0.31)	2.059 (0.19)	2.070 (0.20)
SOS-CC2	2.004 (0.26)	2.012 (0.24)	1.961 (0.29)	2.010 (0.25)	2.154 (0.14)	2.171 (0.14)
SOS-ADC(2)	1.924 (0.29)	1.933 (0.27)	1.879 (0.33)	1.926 (0.28)	2.098 (0.16)	2.116 (0.15)
ωΒ97	1.835 (0.25)	1.829 (0.24)	1.781 (0.28)	1.852 (0.24)	2.026 (0.15)	2.040 (0.15)
ωΒ97X-V	1.907 (0.26)	1.904 (0.24)	1.856 (0.29)	1.916 (0.26)	2.064 (0.17)	2.075 (0.17)
ωB2PLYP	1.881 (0.28)	1.881 (0.27)	1.833 (0.32)	1.885 (0.27)	2.025 (0.17)	2.040 (0.17)
LC-BLYP	1.874 (0.24)	1.868 (0.23)	1.824 (0.27)	1.882 (0.24)	2.017 (0.15)	2.027 (0.15)
CAM-B3LYP	2.000 (0.27)	2.001 (0.25)	1.958 (0.31)	2.000 (0.27)	2.093 (0.20)	2.098 (0.20)
CAM-B3LYP*	2.050 (0.27)	2.055 (0.24)	2.012 (0.30)	2.044 (0.27)	2.104 (0.21)	2.101 (0.21)
B2PLYP	2.033 (0.31)	2.043 (0.28)	1.996 (0.34)	2.018 (0.31)	2.046 (0.22)	2.047 (0.23)
BHandHLYP	2.045 (0.31)	2.048 (0.28)	2.004 (0.34)	2.046 (0.31)	2.147 (0.23)	2.151 (0.24)
B1LYP	2.073 (0.28)	2.080 (0.25)	2.036 (0.31)	2.065 (0.28)	2.119 (0.22)	2.114 (0.22)
B3LYP	2.066 (0.27)	2.073 (0.24)	2.029 (0.29)	2.057 (0.27)	2.101 (0.21)	2.094 (0.21)
PBE0	2.089 (0.28)	2.096 (0.25)	2.051 (0.31)	2.081 (0.28)	2.133 (0.22)	2.129 (0.23)
PBE	1.983 (0.12)	1.997 (0.19)	1.953^b^ (0.24)	1.974^b^ (0.19)	1.903 (0.10)	1.883 (0.08)
BLYP	1.970 (0.13)	1.984 (0.19)	1.941^b^ (0.24)	1.959 (0.11)	1.895 (0.10)	1.873 (0.08)
BP86	1.981 (0.13)	1.996 (0.19)	1.951^b^ (0.24)	1.971 (0.11)	1.903 (0.10)	1.882 (0.09)
ZINDO/S	1.554 (0.38)	1.563 (0.35)	1.524 (0.39)	1.571(0.36)	1.747 (0.17)	1.772 (0.16)

a The electronic excitation energies are reported in the electron volt units (eV) and the oscillator strengths corresponding to the excited state are shown in the parentheses.

b These energies correspond to the second excited state (S_2_); the S_1_ state predicted in these cases is physically incorrect.

**Table 4:** Electrochromic Shifts (in meV) of RC Chromophores upon Electrostatic Embedding, Obtained Using Various Quantum Chemical Methods^a^

method	P_D1_	P_D2_	Chl_D1_	Chl_D2_	Pheo_D1_	Pheo_D2_
DLPNO-STEOM-CCSD	–20	–15	–67	–25	142	177
CC2	11	3	–30	–12	25	15
ADC(2)	33	20	–18	–9	43	34
SCS-CC2	–13	–13	–57	–25	111	119
SCS-ADC(2)	–6	–5	–54	–25	129	141
SOS-CC2	–18	–15	–66	–27	152	167
SOS-ADC(2)	–12	–7	–63	–26	161	177
ωΒ97	–23	–28	–72	–29	166	172
ωΒ97X-V	–18	–23	–64	–27	141	145
ωB2PLYP	–14	–16	–60	–24	152	162
LC-BLYP	–16	–21	–62	–25	136	140
CAM-B3LYP	–12	–13	–49	–22	83	85
CAM-B3LYP*	–5	–3	–38	–16	44	40
B2PLYP	1	2	–36	–15	55	58
BHandHLYP	–14	–14	–49	–22	77	79
B1LYP	–3	–1	–35	–15	34	28
B3LYP	0	1	–33	–13	26	18
PBE0	–2	–1	–35	–15	33	28
PBE	6	7	–18	–5	–82	–101
BLYP	5	7	–18	–8	–79	–100
BP86	5	7	–18	–6	–82	–102
ZINDO/S	–42	–44	–69	–37	132	156

a The electrochromic shift is defined as Q_*y*_ (protein) – Q_*y*_ (gas-phase).

The DLPNO-STEOM-CCSD results show that compared to their gas-phase values, the chlorophyll and pheophytin chromophores are red-shifted and blue-shifted, respectively, upon electrostatic embedding. Chl_D1_ is the chlorophyll that is most affected and displays the greatest red-shift. It also has the highest oscillator strength for the computed S_1_ state among all chromophores. As also reported recently in a multiscale study of the PSII RC,^25^ the electrostatic effect of the protein creates two types of asymmetry: transverse, differentiating the site energies of pheophytins (blue-shift) versus chlorophylls (red-shift), and lateral, differentiating the two branches by localizing the chromophore with the lowest site energy on the D1 branch. This asymmetry of site energies in the RC chromophores is crucial for the D1 branch being active in electron transfer in PSII. Based on our results, Chl_D1_ would form the trap of excitation energy received from the CP43 and CP47 proteins, and is likely the initial electron donor. This would agree with one of the major suggestions in the experimental^23,27,126−128^ and computational^18,120,121^ literature. An alternative scenario^23,26,127^ involves electron donation directly from one or both chlorophylls of the central pair, P_D1_ and P_D2_. The question of the localization and nature of productive RC charge transfer states among groups of chromophores is an independent problem that is essential for deciphering the initial steps of charge separation, but is beyond the scope of the present study.

**Figure 6:**
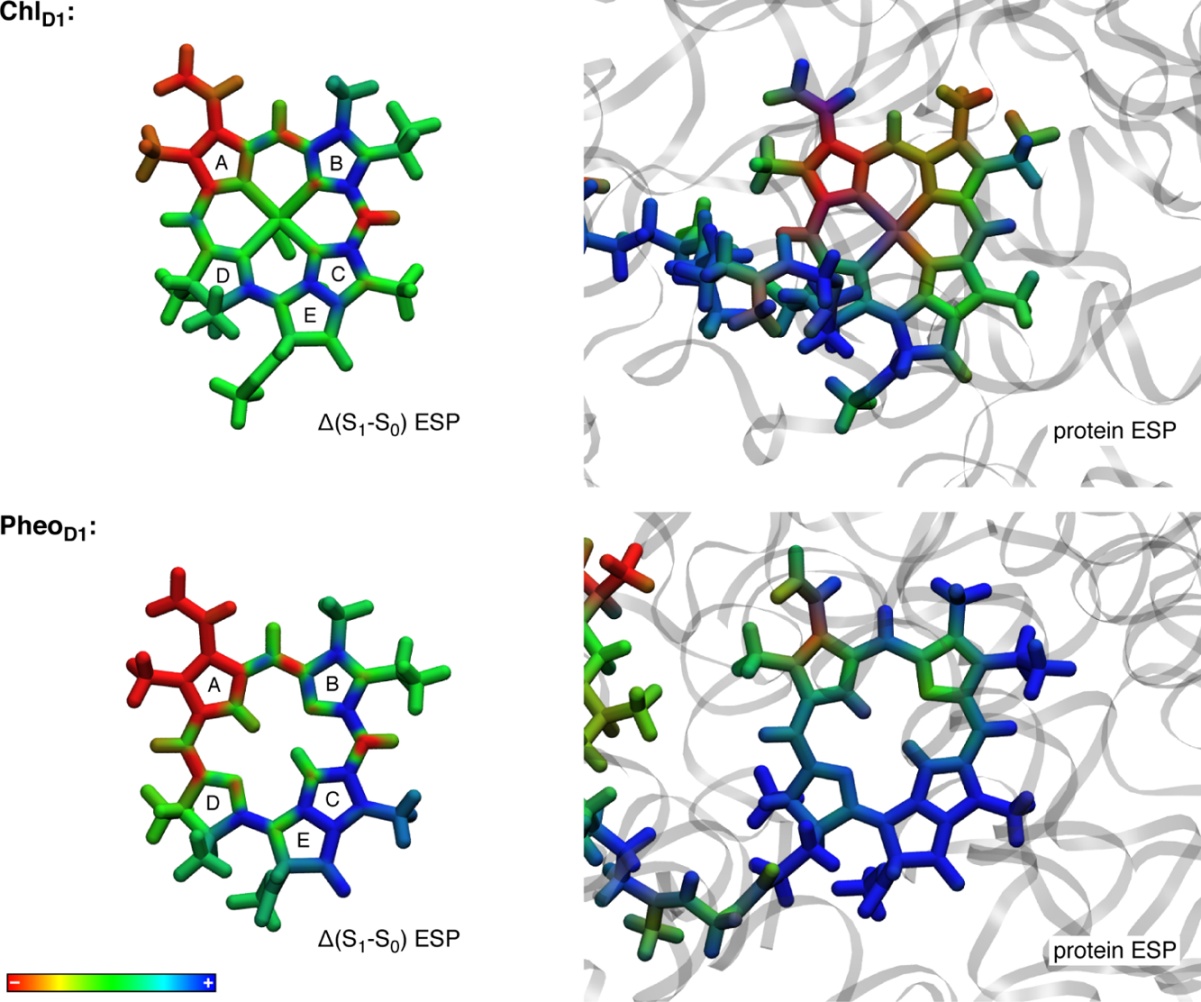
Projections of electrostatic potentials on the molecular frames of the Chl_D1_ and Pheo_D1_ chromophores. Figures on the left depict intrinsic difference ESPs of the chromophores obtained from the difference of first excited state and ground state ESPs. On the right, the electrostatic effect of the protein environment on each chromophore is shown. The scale for the intrinsic ESP maps spans ±300 kT/e and for the protein-induced ESP ±30 kT/e (mV). The intrinsic difference ESP maps were computed at the ωB97X-V/Def2-TZVP level of theory; the influence of protein electrostatics on the chromophores was computed using the adaptive Poisson–Boltzmann solver.^130^

The shifts in excitation energies arise as the difference in the interaction of the ground state and the excited state with the environment. Hence, a blue-shift can come from preferential stabilization of the ground state or destabilization of the excited state or both, and the opposite for a red-shift. Figure 6 compares the intrinsic electrostatic potential difference between the first excited and the ground state of the red-shifted Chl_D1_ and the blue-shifted Pheo_D1_ with the electrostatic potential induced by the protein. For both chromophores, ring A is associated with an increase in the negative electrostatic potential upon excitation, but there is a distinctly different profile for the positive potential difference, weakly distributed in rings B and C for Chl_D1_ but strongly on ring C for Pheo_D1_. In the latter case, the mechanism of protein modulation is to destabilize the excited state, because the same region of the molecule (rings C and E) is under the influence of a strongly electropositive environment. The profile of the electrostatic potential of the protein as projected on rings B and C of the red-shifting Chl_D1_ is distinctly different, essentially neutral for this part of the molecule and with a stabilizing interaction with respect to the R_3_ group. Detailed studies of short- and long-range residue-specific electrostatic effects are discussed in recent literature.^25,129^

Focusing on the numerical results listed in Table 3, it is clear that the absolute values of the lowest vertical excitation energies differ non-negligibly between the different methods. It is noted that the absorption maximum of the PSII RC is around 680 nm (1.82 eV), which is close to the vertical excitation energy computed for Chl_D1_ with LC-BLYP. The absorption maximum and the vertical excitation energy cannot be directly compared, as the absorption features also contain the contribution from the excited vibrational levels. Based on our recent study of the vibronic spectrum of chlorophyll *a*, we found that the gas-phase absorption maximum is red-shifted by ca. 0.05 eV compared to the vertical excitation energy.^83^ Applying the same correction here in a purely empirical manner would suggest that the best agreement in an absolute sense would be with the Chl_D1_ site energy obtained with ωΒ97X-V. In any case, given the sensitivity of absolute values on the choice of method, this type of analysis is not particularly fruitful. It is more important to ensure the correct reproduction of electrochromic shifts (Table 4), as this is the fundamental basis for describing the RC function at a quantum mechanical level and is a feature that cannot be corrected a posteriori if not captured from the outset using a given quantum chemical method. It should also be noted that the choice of functional for geometry optimization is a parameter that can also affect the calculations via the effect on bond length alternation. Here, we have confirmed by comparison of the ωΒ97X-V results obtained for Chl_D1_ with PBE0 and B3LYP optimized geometries that the absolute excitation energies are blue-shifted by ca. 0.05 eV compared to the PBE geometry, but the central parameters discussed in the present work, that is, the electrochromic shifts, are not affected (differences in the order of 1 meV).

From the results listed in Table 4, we observe that the magnitudes of the electrochromic shifts for each chromophore are distinct. Focusing first on the DFT methods, in comparison to DLPNO-STEOM-CCSD, the range-separated functionals (with 100% long-range HF exchange) ωΒ97, LC-BLYP, ωΒ97X-V, and ωB2PLYP clearly outperform other methods. CAM-B3LYP, a popular choice for TD-DFT calculations, surprisingly underestimates the electrochromic shifts, especially for the pheophytins, compared to other range-separated functionals. Interestingly, setting the attenuation parameter to 0.14, as suggested in a recent study,^109^ makes the performance of CAM-B3LYP markedly worse. The original idea behind the suggested tuning of the attenuation parameter was to reproduce the transition dipole moment of the “Q_*y*_” transition and the absorption spectrum of chlorophylls *a* and *b* in an explicit solvent. However, empirical adjustment of functional parameters to improve the numerical results and create tailored solutions for a specific system or property is an unreliable way of “dialing in” case-specific error cancellations. By partially compensating for other methodological deficiencies, it sacrifices transferability and generality, leading to deteriorated performance for other properties or other types of application, as is the case here.

Global hybrids (PBE0, B1LYP, B3LYP, BHandHLYP, and B2PLYP) predict the trends in electrochromic shifts correctly in most cases, but they suffer from heavy quantitative underestimation, especially in the case of pheophytins. Higher percentage of Hartree–Fock exchange in a global hybrid functional (BHandHLYP) systematically improves the prediction of electrochromic shifts, albeit without adequately approximating the reference. The double-hybrid B2PLYP has no advantage over a conventional global hybrid and performs markedly worse than its range-separated variant.

The results obtained with the GGA functionals show a qualitatively flawed trend. P_D1_ and P_D2_ are blue-shifted, whereas Chl_D1_ and Chl_D2_ are only slightly red-shifted. Surprisingly, both pheophytins red-shift upon electrostatic embedding. Unlike other methods, GGA functionals predict Pheo_D2_ to be the most red-shifted chromophore. Moreover, GGA functionals once again show the same trait in predicting the “ghost” first excited states for Chl_D1_ and Chl_D2_, that is, Q_*y*_ is S_2_ rather than S_1_.

**Figure 7:**
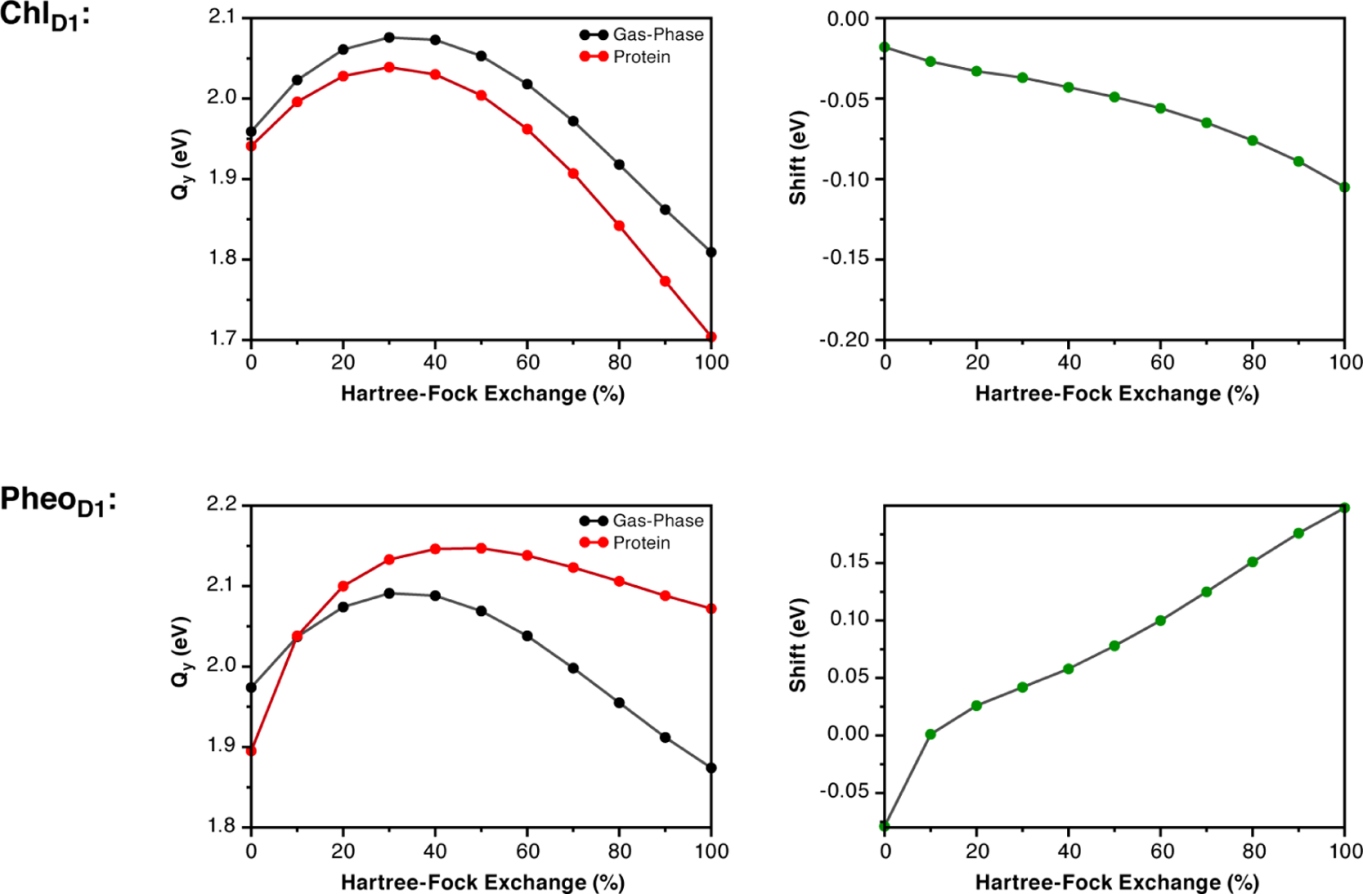
Dependence of the first excitation energy (left) and of the electrochromic shift (right) for Chl_D1_ (top) and Pheo_D1_ (bottom) on the amount of the Hartree–Fock exchange (HFX) in the B1LYP functional.

To examine the dependence of the S_1_ transition energy on the amount of the Hartree–Fock exchange, we chose the two chemically distinct chromophores Chl_D1_ and Pheo_D1_ and studied their excited state properties with QM and QM/MM calculations using the B1LYP functional with variable HF exchange (Figure 7). In the case of Chl_D1_, increasing the amount of HF exchange in the B1LYP functional up to 30% blue-shifts the absolute value of the S_1_ state (Q_*y*_) both in vacuo and in the protein. Interestingly, 40% HF exchange or more results in overall red-shifting of the absolute S_1_ energies. The electrochromic shift for Chl_D1_ is negative throughout the HF range and increases monotonically with increasing HF exchange. In the case of Pheo_D1_, 0% HF exchange yields the red-shifted S_1_ state for protein-embedded Pheo_D1_ with respect to the gas phase, similar to the GGA functionals. Increasing the amount of HF exchange reverses this and the electrochromic shift changes sign past the crossing point of 10% HF exchange. Therefore, a slightly more complex behavior is seen for the pheophytin, but overall we also observe a monotonic change in the electrochromic shift. Crucially, however, in this case the increased amount of HF exchange leads to a greater blue-shift, whereas for Chl_D1_, the same increase leads to a greater red-shift. The fact that only range-separated functionals seem to perform as well as the reference method is suggestive of the fact that no unique value of HF exchange can be considered appropriate for all chromophores and, hence, that global hybrid functionals are simply not applicable to this and analogous problems.

ZINDO/S performs remarkably well, particularly in view of its low computational cost. Although its performance in absolute terms is not particularly convincing because it overestimates the shifts for P_D1_/P_D2_ and underestimates the shifts for both pheophytins, it does perform better than most density functionals, including CAM-B3LYP, in predicting the nature and magnitude of the electrochromic shifts. Similar observations were made in a past study of the Fenna–Matthew–Olson complex, but it was also noted that the method might overestimate the coupling to the environment and thus exaggerate the variations in site energies.^112^

Our final point of analysis concerns the alternative approximations to coupled cluster theory, CC2, ADC(2), and their spin component-scaled variants. This is an important topic because these methods are fast, accessible, and have already found widespread use in quantum chemical studies of biochromophores. The present results for CC2 reveal that the method is severely challenged in providing a qualitatively correct picture of the overall shifts across RC chromophores. It fails to differentiate between pigments (Table 4) and uniformly underestimates electrochromic shifts relative to gas-phase values. It predicts that the P_D1_ and P_D2_ chlorophylls are slightly blue-shifted rather than red-shifted like Chl_D1_ and Chl_D2_, and severely underestimates the pheophytin blue-shift. According to CC2, the low-energy excitonic spectrum would mainly consist of Chl_D1_, Chl_D2_, and Pheo_D2_ contributions, a conclusion that is unphysical. The results obtained with ADC(2) are similar to CC2. These results imply that the excitonic asymmetry in the RC is not correctly captured by CC2 and ADC(2) because these methods do not respond adequately to the electrostatic field of the protein compared to other methods. In part, this might be related to the problematic description of the S_1_ excitation itself (see Supporting Information), the same problem as seen in the gas-phase calculations, but regardless of the origin it is clear that the overall shift and relative site-energy ordering of the chromophores is not accessible with these methods.

The SCS and SOS variants of CC2 and ADC(2) show a clear and definite improvement over canonical CC2 and ADC(2) in the description of the Q_*y*_ excitation itself and in the reproduction of electrochromic shifts. Thus, these are the only members of this family that qualitatively demonstrate the transverse and lateral asymmetry of RC chromophores. The SOS versions of CC2 and ADC(2) provide quantitatively the best results and, at least for this system, are fully in line with the results obtained from DLPNO-STEOM-CCSD and with the best performing range-separated functionals. Precise reasons for the dramatic altered performance of these methods upon scaling of the different spin components are hard to pinpoint, thus the present results are offered as straightforward observations. Although the use of SOS-CC2 or SOS-ADC(2) can be recommended in the present case, the variability of results advocates caution when employing CC2 and ADC(2) or their spin component-scaled variants. These methods should not be considered as reference quality methods without higher-level benchmarking and it is inadvisable to use them for benchmarking (TD)DFT because they may be surpassed by modern range-separated functionals in robustness and reliability.

**Figure 8:**
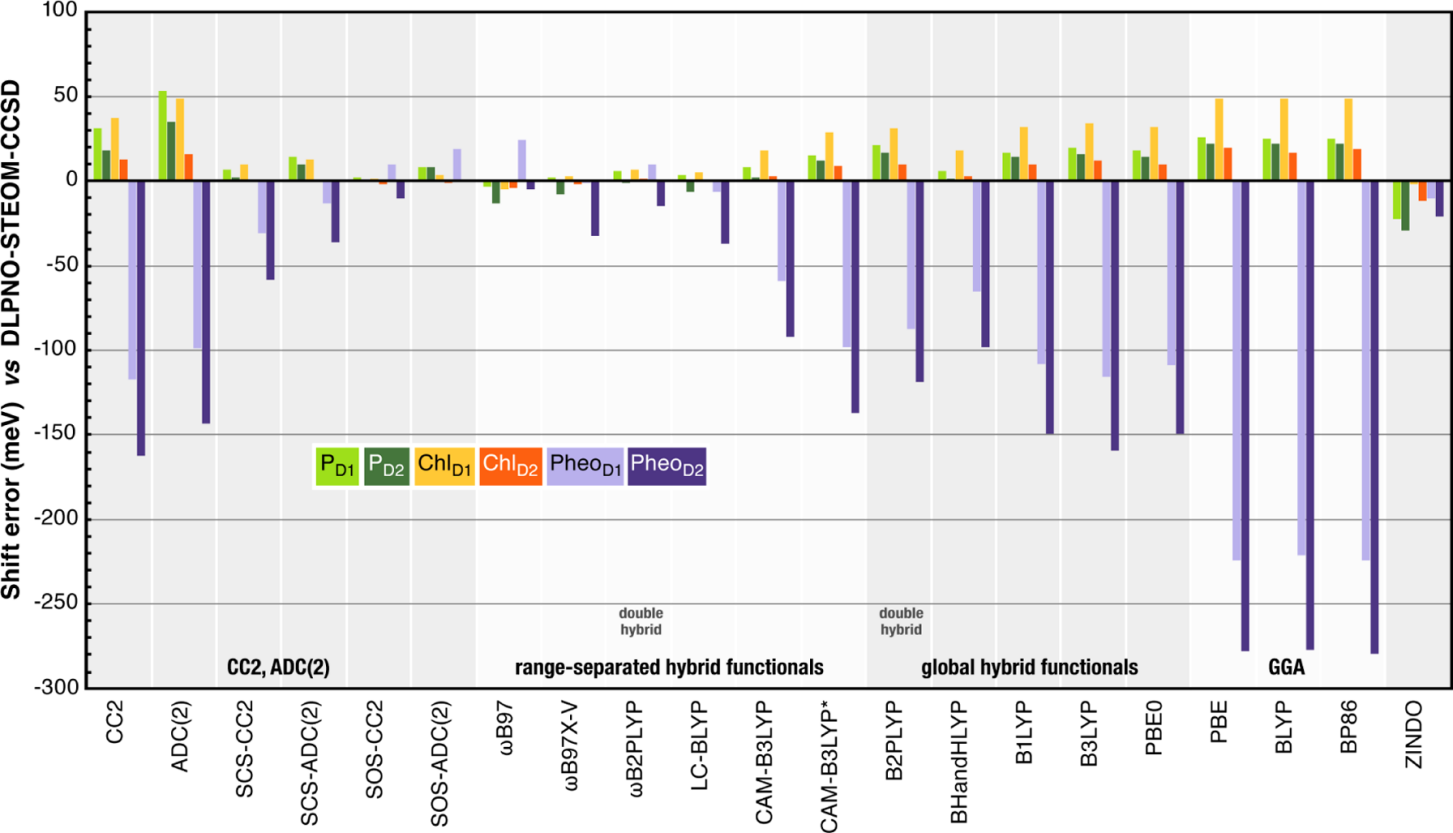
Errors in electrochromic shifts (in meV) for all methods against the DLPNO-STEOM-CCSD reference.

Figure 8 provides an overview of the errors in electrochromic shifts for all methods and pigments using the DLPNO-STEOM-CCSD values as reference.

### 3.3 Comparison of Intact PSII with the RC Complex

An intact PSII complex contains dozens of chlorophyll molecules, which leads to a highly congested absorption spectrum, making the direct investigation of RC photochemistry almost impossible. To reduce this complexity, the study of the RC is generally performed using the PSII-RCC (RC complex) preparation.^131−135^ This retains only the three core proteins (D1–D2–Cyt_b559_) and is considered a minimal structural scaffold. Most of the existing information on the RC of PSII is derived from studies on PSII-RCC preparations. Atomistic structural details of the PSII-RCC are not currently available and, hence, a direct structural comparison with native PSII is impossible. However, we can still use the information from our intact computational PSII model to provide “best case scenario” estimates for the global electrostatic effects on the modulation of site energies of RC chromophores in intact PSII versus a hypothetical structurally unperturbed PSII-RCC. We perform this task by using the same distribution of point charges as with our intact PSII model, deleting all point charges belonging to protein subunits other than D1–D2–Cyt_b559_. All redox-active cofactors belonging to the D1–D2–Cyt_b559_ are retained.

The results of these calculations are summarized in Table 5. We observe that the site energies of P_D1_, Chl_D2_, and Pheo_D2_ are slightly red-shifted in the minimal D1–D2–Cyt_b559_ model, whereas the site energies of the P_D2_, Chl_D1_, and Pheo_D1_ are blue-shifted. However, the effect is small and only exceeds 10 meV (89 cm^–1^) in the case of Chl_D1_. This is the most affected among all RC chromophores by switching off the global electrostatic effect of the other proteins that comprise the complete PSII monomer. Overall, we conclude that the lateral and transverse excitonic asymmetry in the RC pigments is retained in the D1–D2–Cyt_B559_ complex.

**Table 5:** Site Energies (in eV) of the RC Chromophores of the Intact PSII and the Minimal D1–D2–Cyt_b559_ (PSII-RCC) Model^a^

	intact PSII	PSII-RCC	shift
P_D1_	1.907 (0.26)	1.899 (0.26)	0.008
P_D2_	1.904 (0.24)	1.913 (0.25)	–0.009
Chl_D1_	1.856 (0.29)	1.867 (0.28)	–0.011
Chl_D2_	1.916 (0.26)	1.911 (0.25)	0.005
Pheo_D1_	2.064 (0.17)	2.068 (0.17)	–0.004
Pheo_D2_	2.075 (0.17)	2.074 (0.17)	0.001

a Oscillator strengths are shown in parentheses. All computations are performed using the ωB97X-V/Def2-TZVP level of theory. The shift [Q_*y*_ (intact PSII) – Q_*y*_ (PSII-RCC)], in eV, represents the role of global electrostatics from the intrinsic antenna complexes and the extrinsic proteins in modulating the site energies.

The present results represent the lower limits for PSII-RCC shifts because they are based on the following assumptions: (1) the overall organization and conformation of PSII-RCC is almost the same as that of intact PSII, (2) the orientation of the chromophores with respect to the membrane plane remains the same, (3) no new water channels are formed, (4) no drastic changes occur in the protonation state of residues in the transmembrane region, and (5) all redox-active cofactors remain intact. Although the real structure of PSII-RCC is unknown, we do know that the OEC is missing or nonfunctional (one of the key ligands to the OEC is CP43-Glu 354 and there is a critical CP43-Arg 357 residue in the second coordination sphere), Y_Z_ and Y_D_ do not function as in the native system,^136^ while on the acceptor side both plastoquinones (Q_A_ and Q_B_) might be lost depending on sample preparation.^137^ It has also been shown^132,138^ that certain spectroscopic features of the PSII-CC are missing in the PSII-RCC. Therefore, we consider almost certain that most if not all of the above assumptions do not hold in reality, and hence the differences in site energies can be much larger than the “best case scenario” values reported above. The extent of the differences would have important implications regarding the extent to which PSII-RCC preparations are representative of physiological PSII. This issue might be resolved if more direct structural information on PSII-RCC becomes available. Nevertheless, the present analysis demonstrates that the electrostatic effects that create the asymmetry in excitation energies^18^ within the RC derive almost exclusively from the core D1, D2, and Cyt_b559_ proteins, regardless of whether achieving the correct spatial charge distribution in these core components requires the full complement of PSII proteins.

## 4 Conclusions

We presented an extensive evaluation of theoretical methods for the prediction of electrochromic shifts in the RC chromophores of PSII, defined as the electrostatic effect of the protein on the site energies of individual chromophores compared to gas-phase values. The results first of all highlight the fact that protein matrix effects are explicitly required to study the excited state properties of photosynthetic chromophores because protein electrostatics are exclusively responsible for creating excitonic asymmetry within the RC. Therefore, the QM/MM technique, or any other higher-level methodology that considers explicitly the environment of the pigments, is not a “luxury” but a necessity. It is noted that the present approach considers the simplest possible electrostatic embedding, using a distribution of point charges that influence the QM region. More refined approaches should consider the polarization of the environment,^29,139,140^ a mutual interaction that may affect the numerical values reported here. In addition, the effect of dynamic disorder should be considered when modeling such systems. However, it is expected that the leading effects are already captured at the present level and it is stressed that the performance of the different methods can be adequately evaluated regardless of these aspects. The excitonic asymmetry in the PSII RC is shown to be retained in the D1–D2–Cyt_b559_ complex, provided that the overall protein and cofactor arrangement is assumed to remain the same as the intact PSII core complex.

Among the methods evaluated with respect to reproducing the protein-induced electrochromic shifts, ZINDO deserves a notable mention because it outperforms all standard functionals that do not employ range separation, and even some that do. GGA functionals are unable to describe correctly the low-energy excitations of RC chromophores, therefore they should not be considered applicable to this problem. Global hybrid and double-hybrid functionals manage to capture trends qualitatively for most chromophores, but the produced electrochromic shifts are so severely underestimated that the results are not useful. Range-separated density functionals are the only methods that correctly approximate the reference electrochromic shifts. However, not all of these functionals perform equally well. The range-separated double-hybrid ωΒ2PLYP best matches the DLPNO-STEOM-CCSD values, followed closely by ωΒ97X-V, LC-BLYP, and ωB97. Importantly, CAM-B3LYP does not perform similarly well, either in its standard form or with one of the recommended adjustments, which leads to even worse performance.

Among the tested wave function-based methods, CC2 and ADC(2) in their standard formulations do not describe the Q_*y*_ excitation of chlorophylls convincingly and have severe problems in producing accurate electrochromic shifts. However, a drastic improvement is observed with the SCS and even more so with the SOS versions of these methods for the system and property under consideration. Nevertheless, these results suggest that CC2 and ADC(2) should not be blindly considered as benchmark-quality methods that can be safely used for obtaining reference results or for evaluating DFT. In the present case, they are neither quantitatively nor (for the canonical versions) qualitatively better than many range-separated functionals. This point becomes more critical if the ultimate target is the description of charge-transfer excitations, inherently challenging for CC2,^44,141^ but which are highly relevant for photosynthetic systems.

The methodological conclusions reached in the present work are expected to be valid for any kind of electrochromic/Stark effect that involves the RC of PSII. This includes, for example, understanding the modulation of site energies upon changes in the redox and protonation states of plastoquinones Q_A_ and Q_B_, the redox-active tyrosines, the OEC, and the depletion or substitution of specific ions, etc. It is furthermore expected that the conclusions are directly transferable to any similar system, such as the study of excited state properties of chromophores in the intrinsic antennae (CP43 and CP47), in light harvesting complexes (LHCI and LHCII), the FMO complex, etc. We hope that the present results will inspire and guide more reliable, more accurate, and more insightful QM/MM studies of biochromophores.

## Supporting Information Available

Vertical excitation energies of the lowest excited state (i.e., Q*_y_*) using the QM/MM optimized geometries of the RC chromophores computed in the gas phase and in the protein electrostatic environment using canonical CC2, SOS-CC2, SCS-CC2,ADC(2), SOS-ADC(2), SCS-ADC(2), and DLPNO-STEOM-CCSD; vertical excitation energies of the lowest excited state (i.e., Q*_y_*) using the gas-phase optimized geometry (at the PBE/Def2-TZVP level of theory) of Chlorophyll *a* computed using DLPNO-STEOM-CCSD, CC2, ADC(2), SOS-CC2, SOSADC(2), SCS-CC2, and SCS-ADC(2); and Cartesian coordinates of QM/MM-optimized RC chromophores (PDF)
