# Supplementary material for: How Can We Predict Accurate Electrochromic Shifts for Biochromophores? A Case Study on the Photosynthetic Reaction Center
Source: J Chem Theory Comput. 2021 Feb 10;17(3):1858–73. doi: 10.1021/acs.jctc.0c01152 (PMC8023663; doi:10.1021/acs.jctc.0c01152)
Supplement: Supplementary file 1 — ct0c01152_si_001.pdf [file ct0c01152_si_001.pdf]

How can we predict accurate electrochromic shifts for biochromophores? A case study on the photosynthetic reaction center

Abhishek Sirohiwal,[ab] Frank Neese,[a] Dimitrios A. Pantazis[a]*

[a] Max-Planck-Institut für Kohlenforschung, Kaiser-Wilhelm-Platz 1, 45470 Mülheim an der Ruhr, Germany.

[b] Fakultät für Chemie und Biochemie, Ruhr-Universität Bochum, 44780 Bochum, Germany

Supporting Information

Table S1. The vertical excitation energies of the lowest excited state (i.e. Q_y) using the QM/MM optimized geometries of the RC chromophores computed using canonical CC2 in the gas phase. Corresponding oscillator strengths (*f*) are also shown. The major contributions in the canonical basis associated with the Q_y state are described.

Chromophore	Energy (eV)	*f*	Frontier Molecular Orbitals	% Contribution
P_{D1}	2.126	0.19	HOMO → LUMO<br>HOMO−1 → LUMO<br>HOMO−1 → LUMO+1	0.50<br>0.27<br>0.15
P_{D2}	2.131	0.16	HOMO → LUMO<br>HOMO−1 → LUMO<br>HOMO−1 → LUMO+1	0.44<br>0.32<br>0.13
Chl_{D1}	2.128	0.21	HOMO → LUMO<br>HOMO−1 → LUMO<br>HOMO−1 → LUMO+1	0.51<br>0.26<br>0.14
Chl_{D2}	2.117	0.21	HOMO → LUMO<br>HOMO−1 → LUMO<br>HOMO−1 → LUMO+1	0.53<br>0.24<br>0.15
$Pheo_{D1}$	2.089	0.17	HOMO → LUMO<br>HOMO−1 → LUMO+1	0.69<br>0.25
$Pheo_{D2}$	2.084	0.17	HOMO → LUMO<br>HOMO−1 → LUMO+1	0.69<br>0.25

Table S2. The vertical excitation energies of the lowest excited state (i.e. Q_y) using the QM/MM optimized geometries of the RC chromophores computed using canonical CC2 in the protein electrostatic environment. Corresponding oscillator strengths (*f*) are also shown. The major contributions in the canonical basis associated with the Q_y state are described.

Chromophore	Energy (eV)	*f*	Frontier Molecular Orbitals	% Contribution
P_{D1}	2.137	0.25	HOMO → LUMO<br>HOMO−1 → LUMO<br>HOMO−1 → LUMO+1	0.60<br>0.18<br>0.15
P_{D2}	2.134	0.20	HOMO → LUMO<br>HOMO−1 → LUMO<br>HOMO−1 → LUMO+1	0.54<br>0.24<br>0.14
Chl_{D1}	2.098	0.27	HOMO → LUMO<br>HOMO−1 → LUMO<br>HOMO−1 → LUMO+1	0.56<br>0.23<br>0.14
Chl_{D2}	2.105	0.25	HOMO → LUMO<br>HOMO−1 → LUMO<br>HOMO−1 → LUMO+1	0.60<br>0.18<br>0.15
$Pheo_{D1}$	2.114	0.19	HOMO → LUMO<br>HOMO−1 → LUMO+1	0.66<br>0.22
$Pheo_{D2}$	2.099	0.18	HOMO → LUMO<br>HOMO−1 → LUMO+1	0.61<br>0.21

Table S3. The vertical excitation energies of the lowest excited state (i.e. Q_y) using the QM/MM optimized geometries of the RC chromophores computed using SOS-CC2 in the gas phase. Corresponding oscillator strengths (*f*) are also shown. The major contributions in the canonical basis associated with the Q_y state are described.

Chromophore	Energy (eV)	*f*	Frontier Molecular Orbitals	% Contribution
P_{D1}	2.022	0.21	HOMO → LUMO<br>HOMO−1 → LUMO+1	0.72<br>0.19
P_{D2}	2.027	0.19	HOMO → LUMO<br>HOMO−1 → LUMO+1	0.72<br>0.21
Chl_{D1}	2.027	0.23	HOMO → LUMO<br>HOMO−1 → LUMO+1	0.73<br>0.19
Chl_{D2}	2.037	0.22	HOMO → LUMO<br>HOMO−1 → LUMO+1	0.72<br>0.19
$Pheo_{D1}$	2.002	0.17	HOMO → LUMO<br>HOMO−1 → LUMO+1	0.69<br>0.24
$Pheo_{D2}$	2.004	0.17	HOMO → LUMO<br>HOMO−1 → LUMO+1	0.69<br>0.24

Table S4. The vertical excitation energies of the lowest excited state (i.e. Q_y) using the QM/MM optimized geometries of the RC chromophores computed using SOS-CC2 in the protein electrostatic environment. Corresponding oscillator strengths (*f*) are also shown. The major contributions in the canonical basis associated with the Q_y state are described.

Chromophore	Energy (eV)	*f*	Frontier Molecular Orbitals	% Contribution
P_{D1}	2.004	0.26	HOMO → LUMO<br>HOMO−1 → LUMO+1	0.76<br>0.18
P_{D2}	2.012	0.24	HOMO → LUMO<br>HOMO−1 → LUMO+1	0.76<br>0.19
Chl_{D1}	1.961	0.29	HOMO → LUMO<br>HOMO−1 → LUMO+1	0.77<br>0.17
Chl_{D2}	2.010	0.25	HOMO → LUMO<br>HOMO−1 → LUMO+1	0.75<br>0.18
$Pheo_{D1}$	2.154	0.14	HOMO → LUMO<br>HOMO−1 → LUMO+1	0.68<br>0.23
$Pheo_{D2}$	2.171	0.14	HOMO → LUMO<br>HOMO−1 → LUMO+1	0.67<br>0.24

Table S5. The vertical excitation energies of the lowest excited state (i.e. Q_y) using the QM/MM optimized geometries of the RC chromophores computed using SCS-CC2 in the gas phase. Corresponding oscillator strengths (*f*) are also shown. The major contributions in the canonical basis associated with the Q_y state are described.

Chromophore	Energy (eV)	*f*	Frontier Molecular Orbitals	% Contribution
P_{D1}	2.064	0.23	HOMO → LUMO<br>HOMO−1 → LUMO+1	0.75<br>0.20
P_{D2}	2.071	0.20	HOMO → LUMO<br>HOMO−1 → LUMO+1	0.74<br>0.21
Chl_{D1}	2.068	0.24	HOMO → LUMO<br>HOMO−1 → LUMO+1	0.75<br>0.19
Chl_{D2}	2.071	0.24	HOMO → LUMO<br>HOMO−1 → LUMO+1	0.75<br>0.20
$Pheo_{D1}$	2.023	0.17	HOMO → LUMO<br>HOMO−1 → LUMO+1	0.71<br>0.25
$Pheo_{D2}$	2.023	0.17	HOMO → LUMO<br>HOMO−1 → LUMO+1	0.71<br>0.25

Table S6. The vertical excitation energies of the lowest excited state (i.e. Q_y) using the QM/MM optimized geometries of the RC chromophores computed using SCS-CC2 in the protein electrostatic environment. Corresponding oscillator strengths (*f*) are also shown. The major contributions in the canonical basis associated with the Q_y state are described.

Chromophore	Energy (eV)	*f*	Frontier Molecular Orbitals	% Contribution
P_{D1}	2.051	0.27	HOMO → LUMO<br>HOMO−1 → LUMO+1	0.77<br>0.18
P_{D2}	2.058	0.24	HOMO → LUMO<br>HOMO−1 → LUMO+1	0.76<br>0.19
Chl_{D1}	2.011	0.30	HOMO → LUMO<br>HOMO−1 → LUMO+1	0.78<br>0.17
Chl_{D2}	2.046	0.27	HOMO → LUMO<br>HOMO−1 → LUMO+1	0.77<br>0.18
$Pheo_{D1}$	2.134	0.17	HOMO → LUMO<br>HOMO−1 → LUMO+1	0.71<br>0.24
$Pheo_{D2}$	2.142	0.17	HOMO → LUMO<br>HOMO−1 → LUMO+1	0.71<br>0.24

Table S7. The vertical excitation energies of the lowest excited state (i.e. Q_y) using the QM/MM optimized geometries of the RC chromophores computed using ADC(2) in the gas phase. Corresponding oscillator strengths (f) are also shown. The major contributions in the canonical basis associated with the Q_y state are described.

Chromophore	Energy (eV)	f	Frontier Molecular Orbitals	% Contribution
P_{D1}	1.887	0.21	HOMO → LUMO<br>HOMO−1 → LUMO<br>HOMO−1 → LUMO+1	0.46<br>0.37<br>0.07
P_{D2}	1.898	0.18	HOMO → LUMO<br>HOMO−1 → LUMO<br>HOMO→ LUMO+1	0.45<br>0.36<br>0.07
Chl_{D1}	1.896	0.24	HOMO → LUMO<br>HOMO−1 → LUMO<br>HOMO−1 → LUMO+1	0.51<br>0.32<br>0.08
Chl_{D2}	1.883	0.25	HOMO → LUMO<br>HOMO−1 → LUMO<br>HOMO−1 → LUMO+1	0.57<br>0.26<br>0.09
$Pheo_{D1}$	1.873	0.23	HOMO → LUMO<br>HOMO−1 → LUMO+1	0.74<br>0.17
$Pheo_{D2}$	1.863	0.23	HOMO → LUMO<br>HOMO−1 → LUMO+1	0.74<br>0.16

Table S8. The vertical excitation energies of the lowest excited state (i.e. Q_y) using the QM/MM optimized geometries of the RC chromophores computed using ADC(2) in the protein electrostatic environment. Corresponding oscillator strengths (f) are also shown. The major contributions in the canonical basis associated with the Q_y state are described.

Chromophore	Energy (eV)	f	Frontier Molecular Orbitals	% Contribution
P_{D1}	1.920	0.28	HOMO → LUMO<br>HOMO−1 → LUMO<br>HOMO−1 → LUMO+1	0.62<br>0.22<br>0.09
P_{D2}	1.918	0.25	HOMO → LUMO<br>HOMO−1 → LUMO<br>HOMO−1 → LUMO+1	0.59<br>0.24<br>0.08
Chl_{D1}	1.878	0.29	HOMO → LUMO<br>HOMO−1 → LUMO<br>HOMO−1 → LUMO+1	0.53<br>0.31<br>0.08
Chl_{D2}	1.874	0.29	HOMO → LUMO<br>HOMO−1 → LUMO<br>HOMO−1 → LUMO+1	0.65<br>0.19<br>0.09
$Pheo_{D1}$	1.916	0.23	HOMO → LUMO<br>HOMO−1 → LUMO+1	0.72<br>0.14
$Pheo_{D2}$	1.897	0.22	HOMO → LUMO<br>HOMO−1 → LUMO<br>HOMO−1 → LUMO+1	0.67<br>0.14<br>0.13

Table S9. The vertical excitation energies of the lowest excited state (i.e. Q_y) using the QM/MM optimized geometries of the RC chromophores computed using SOS-ADC(2) in the gas phase. Corresponding oscillator strengths (*f*) are also shown. The major contributions in the canonical basis associated with the Q_y state are described.

Chromophore	Energy (eV)	*f*	Frontier Molecular Orbitals	% Contribution
P_{D1}	1.936	0.24	HOMO → LUMO<br>HOMO−1 → LUMO+1	0.73<br>0.16
P_{D2}	1.940	0.21	HOMO → LUMO<br>HOMO−1 → LUMO+1	0.73<br>0.18
Chl_{D1}	1.942	0.26	HOMO → LUMO<br>HOMO−1 → LUMO+1	0.74<br>0.16
Chl_{D2}	1.952	0.24	HOMO → LUMO<br>HOMO−1 → LUMO+1	0.73<br>0.16
$Pheo_{D1}$	1.937	0.19	HOMO → LUMO<br>HOMO−1 → LUMO+1	0.72<br>0.21
$Pheo_{D2}$	1.939	0.19	HOMO → LUMO<br>HOMO−1 → LUMO+1	0.72<br>0.21

Table S10. The vertical excitation energies of the lowest excited state (i.e. Q_y) using the QM/MM optimized geometries of the RC chromophores computed using SOS-ADC(2) in the protein electrostatic environment. Corresponding oscillator strengths (*f*) are also shown. The major contributions in the canonical basis associated with the Q_y state are described.

Chromophore	Energy (eV)	*f*	Frontier Molecular Orbitals	% Contribution
P_{D1}	1.924	0.29	HOMO → LUMO<br>HOMO−1 → LUMO+1	0.78<br>0.15
P_{D2}	1.933	0.27	HOMO → LUMO<br>HOMO−1 → LUMO+1	0.79<br>0.16
Chl_{D1}	1.879	0.33	HOMO → LUMO<br>HOMO−1 → LUMO+1	0.79<br>0.14
Chl_{D2}	1.926	0.28	HOMO → LUMO<br>HOMO−1 → LUMO+1	0.77<br>0.15
$Pheo_{D1}$	2.098	0.16	HOMO → LUMO<br>HOMO−1 → LUMO+1	0.69<br>0.21
$Pheo_{D2}$	2.116	0.15	HOMO → LUMO<br>HOMO−1 → LUMO+1	0.67<br>0.21

Table S11. The vertical excitation energies of the lowest excited state (i.e. Q_y) using the QM/MM optimized geometries of the RC chromophores computed using SCS-ADC(2) in the gas phase. Corresponding oscillator strengths (*f*) are also shown. The major contributions in the canonical basis associated with the Q_y state are described.

Chromophore	Energy (eV)	*f*	Frontier Molecular Orbitals	% Contribution
P_{D1}	1.946	0.26	HOMO → LUMO<br>HOMO−1 → LUMO+1	0.77<br>0.16
P_{D2}	1.952	0.23	HOMO → LUMO<br>HOMO−1 → LUMO+1	0.77<br>0.18
Chl_{D1}	1.952	0.28	HOMO → LUMO<br>HOMO−1 → LUMO+1	0.78<br>0.16
Chl_{D2}	1.955	0.27	HOMO → LUMO<br>HOMO−1 → LUMO+1	0.78<br>0.16
$Pheo_{D1}$	1.930	0.21	HOMO → LUMO<br>HOMO−1 → LUMO+1	0.75<br>0.20
$Pheo_{D2}$	1.929	0.21	HOMO → LUMO<br>HOMO−1 → LUMO+1	0.75<br>0.20

Table S12. The vertical excitation energies of the lowest excited state (i.e. Q_y) using the QM/MM optimized geometries of the RC chromophores computed using SCS-ADC(2) in the protein electrostatic environment. Corresponding oscillator strengths (*f*) are also shown. The major contributions in the canonical basis associated with the Q_y state are described.

Chromophore	Energy (eV)	*f*	Frontier Molecular Orbitals	% Contribution
P_{D1}	1.940	0.31	HOMO → LUMO<br>HOMO−1 → LUMO+1	0.81<br>0.14
P_{D2}	1.947	0.29	HOMO → LUMO<br>HOMO−1 → LUMO+1	0.80<br>0.15
Chl_{D1}	1.898	0.35	HOMO → LUMO<br>HOMO−1 → LUMO+1	0.81<br>0.14
Chl_{D2}	1.930	0.31	HOMO → LUMO<br>HOMO−1 → LUMO+1	0.81<br>0.14
$Pheo_{D1}$	2.059	0.19	HOMO → LUMO<br>HOMO−1 → LUMO+1	0.75<br>0.20
$Pheo_{D2}$	2.070	0.20	HOMO → LUMO<br>HOMO−1 → LUMO+1	0.75<br>0.20

Table S13. The vertical excitation energies of the lowest excited state (i.e. Q_y) using the QM/MM optimized geometries of the RC chromophores computed using DLPNO-STEOM-CCSD in the gas phase. Corresponding oscillator strengths (*f*) are also shown. The major contributions in the canonical basis associated with the Q_y state are described.

Chromophore	Energy (eV)	*f*	Frontier Molecular Orbitals	% Contribution
P_{D1}	1.633	0.22	HOMO → LUMO<br>HOMO−1 → LUMO+1	0.69<br>0.18
P_{D2}	1.635	0.20	HOMO → LUMO<br>HOMO−1 → LUMO+1	0.69<br>0.19
Chl_{D1}	1.642	0.23	HOMO → LUMO<br>HOMO−1 → LUMO+1	0.69<br>0.17
Chl_{D2}	1.649	0.23	HOMO → LUMO<br>HOMO−1 → LUMO+1	0.68<br>0.17
$Pheo_{D1}$	1.601	0.15	HOMO → LUMO<br>HOMO−1 → LUMO+1	0.66<br>0.25
$Pheo_{D2}$	1.591	0.16	HOMO → LUMO<br>HOMO−1 → LUMO+1	0.66<br>0.25

Table S14. The vertical excitation energies of the lowest excited state (i.e. Q_y) using the QM/MM optimized geometries of the RC chromophores computed using DLPNO-STEOM-CCSD in the protein electrostatic environment. Corresponding oscillator strengths (*f*) are also shown. The major contributions in the canonical basis associated with the Q_y state are described.

Chromophore	Energy (eV)	*f*	Frontier Molecular Orbitals	% Contribution
P_{D1}	1.613	0.27	HOMO → LUMO<br>HOMO−1 → LUMO+1	0.73<br>0.17
P_{D2}	1.620	0.26	HOMO → LUMO<br>HOMO−1 → LUMO+1	0.74<br>0.18
Chl_{D1}	1.575	0.30	HOMO → LUMO<br>HOMO−1 → LUMO+1	0.74<br>0.16
Chl_{D2}	1.624	0.26	HOMO → LUMO<br>HOMO−1 → LUMO+1	0.71<br>0.17
$Pheo_{D1}$	1.743	0.13	HOMO → LUMO<br>HOMO−1 → LUMO+1	0.63<br>0.26
$Pheo_{D2}$	1.768	0.12	HOMO → LUMO<br>HOMO−1 → LUMO+1	0.62<br>0.27

Table S15. The vertical excitation energies of the lowest excited state (i.e. Q_y) using gas-phase optimized geometry of the Chlorophyll *a* computed using DLPNO-STEOM-CCSD, CC2, ADC(2), SOS-CC2, SOS-ADC(2), SCS-CC2 and SCS-ADC(2) The geometry in this case is optimized at the PBE/Def2-TZVP level of theory. Corresponding oscillator strengths (*f*) are also shown. The major contributions in the canonical basis associated with the Q_y state are described. The coordinates for this geometry are provided at the end of this document.

Method	Energy (eV)	*f*	Frontier Molecular Orbitals	% Contribution
DLPNO-STEOM-CCSD	1.663	0.25	HOMO → LUMO<br>HOMO−1 → LUMO+1	0.72<br>0.19
CC2	2.146	0.25	HOMO → LUMO<br>HOMO−1 → LUMO+1	0.70<br>0.16
SOS-CC2	2.021	0.23	HOMO → LUMO<br>HOMO−1 → LUMO+1	0.75<br>0.19
SCS-CC2	2.064	0.24	HOMO → LUMO<br>HOMO−1 → LUMO+1	0.77<br>0.18
ADC(2)	1.925	0.29	HOMO → LUMO<br>HOMO−1 → LUMO+1	0.74<br>0.11
SOS-ADC(2)	1.936	0.26	HOMO → LUMO<br>HOMO−1 → LUMO+1	0.77<br>0.16
SCS-ADC(2)	1.947	0.28	HOMO → LUMO<br>HOMO−1 → LUMO+1	0.79<br>0.15

QM/MM Optimized Geometries of Reaction Center Chromophores

1. P_{D1}

C	6.561183000	-1.337207000	-2.286065000
H	6.676942000	-2.364582000	-1.916161000
H	7.192958000	-0.675062000	-1.679021000
C	5.124996000	-0.930653000	-2.166373000
N	4.737416000	0.371020000	-1.917928000
H	5.334816000	1.193472000	-1.770921000
C	3.389192000	0.425031000	-1.875317000
H	2.827933000	1.336427000	-1.696338000
N	2.868858000	-0.780775000	-2.076528000
C	3.941222000	-1.634565000	-2.262778000
H	3.799038000	-2.696824000	-2.434626000
C	-0.885181000	-4.449801000	-5.625559000
C	-1.464510000	3.906245000	-2.693646000
C	-0.134366000	-0.520158000	3.781782000
C	1.345966000	-6.491047000	-1.663925000
N	0.590609000	-2.225264000	-3.815376000
C	-2.090227000	4.805988000	-3.480338000
C	-1.534225000	-0.497010000	4.415842000
C	1.289603000	-5.959793000	-3.192419000
N	-0.149696000	0.447149000	-2.755974000
O	1.368412000	-7.673442000	-1.372947000
C	2.533595000	-6.436934000	-3.879334000
N	0.892038000	-3.042951000	-0.990643000
C	3.468203000	-8.392990000	-4.846346000
C	1.092565000	-4.465090000	-3.066586000
C	0.031510000	-0.230275000	-5.115860000
C	-0.653484000	1.691347000	-0.701512000
C	0.679127000	-2.497829000	1.386841000
C	2.029951000	-2.208749000	-6.780757000
C	-0.911119000	2.707585000	-5.636723000
C	-0.986864000	2.165166000	2.377600000
C	1.404911000	-5.603695000	1.724024000
C	0.807684000	-3.553185000	-4.062310000
C	-0.250831000	0.705447000	-4.095788000
C	-0.333754000	0.732623000	0.276139000
C	0.943103000	-3.412265000	0.352918000
O	3.597154000	-5.842865000	-3.974717000
C	0.539856000	-3.875315000	-5.518624000
C	-0.728786000	2.052229000	-4.313443000
C	-0.501022000	0.923575000	1.706183000
C	1.211036000	-4.837415000	0.471160000
O	2.328239000	-7.696585000	-4.328013000
C	0.649650000	-2.482430000	-6.173726000
C	-0.948447000	2.605389000	-3.055143000
C	-0.119818000	-0.256414000	2.308782000
C	1.284979000	-5.297954000	-0.847445000
C	0.390746000	-1.563092000	-4.983081000
C	-0.569931000	1.577261000	-2.089473000
C	0.280533000	-1.158533000	1.231308000
C	1.102223000	-4.162249000	-1.687077000

N	0.150668000	-0.519906000	0.012120000
Mg	0.787417000	-1.199088000	-1.884403000
H	-1.593067000	-3.662074000	-5.329312000
H	-1.009769000	-5.279062000	-4.915586000
H	-1.364020000	4.164735000	-1.637540000
H	0.481549000	0.253682000	4.270959000
H	0.349765000	-1.477150000	4.005600000
H	-2.314936000	4.612484000	-4.527375000
H	-2.470206000	5.735033000	-3.056189000
H	-2.151667000	-1.339696000	4.080028000
H	-2.076345000	0.425138000	4.167933000
H	-1.458426000	-0.547081000	5.508421000
H	0.452681000	-6.481441000	-3.679642000
H	3.565982000	-8.201122000	-5.923682000
H	3.269699000	-9.454939000	-4.657145000
H	4.382943000	-8.076823000	-4.336094000
H	-0.092012000	0.128887000	-6.138455000
H	-1.073893000	2.620669000	-0.318842000
H	0.739744000	-2.869980000	2.411686000
H	2.240229000	-2.895762000	-7.615656000
H	2.814281000	-2.333108000	-6.019037000
H	2.079534000	-1.178781000	-7.157276000
H	-0.431221000	2.139405000	-6.445619000
H	-0.473863000	3.715851000	-5.626742000
H	-1.976911000	2.822468000	-5.892988000
H	-0.828425000	2.119285000	3.461476000
H	-2.060827000	2.330273000	2.203467000
H	-0.465049000	3.057866000	2.008495000
H	0.677545000	-6.422478000	1.828803000
H	1.325476000	-4.940843000	2.590446000
H	2.396366000	-6.079407000	1.745880000
H	1.267884000	-4.593403000	-5.927997000
H	-0.119998000	-2.338501000	-6.950339000
H	-1.140234000	-4.800848000	-6.625450000
H	6.946715000	-1.360860000	-3.305316000

2. P_{D2}

C	-11.022748000	3.671999000	-1.950795000
H	-11.272523000	4.612080000	-1.450057000
H	-11.465349000	2.864540000	-1.351235000
C	-9.535578000	3.487269000	-2.025157000
N	-8.934424000	2.340769000	-1.557392000
H	-9.428578000	1.509355000	-1.209747000
C	-7.607336000	2.413907000	-1.788479000
H	-6.900746000	1.638932000	-1.508996000
N	-7.303074000	3.564213000	-2.386263000
C	-8.498963000	4.240367000	-2.543647000
H	-8.534807000	5.228216000	-2.995544000
C	-4.552423000	7.622774000	-6.676039000
C	-3.182927000	-1.051278000	-3.785480000
C	-4.729078000	3.034200000	2.882779000
C	-6.010874000	9.287992000	-2.231040000
N	-5.332948000	5.114555000	-4.595771000
C	-3.606767000	-2.232829000	-4.259697000

C	-6.111074000	3.631075000	3.218910000
C	-6.126400000	8.796863000	-3.764532000
N	-4.518827000	2.400809000	-3.695798000
O	-6.173567000	10.442556000	-1.882883000
C	-7.529239000	9.021535000	-4.272106000
N	-5.167708000	5.877749000	-1.742130000
C	-8.980742000	10.557227000	-5.321535000
C	-5.744971000	7.328226000	-3.722727000
C	-4.871547000	3.160792000	-6.004542000
C	-4.009101000	1.041241000	-1.705535000
C	-4.840103000	5.231743000	0.592056000
C	-7.188371000	4.977407000	-7.362152000
C	-3.685786000	0.306774000	-6.681799000
C	-3.972711000	0.305653000	1.341257000
C	-5.297004000	8.370476000	1.081159000
C	-5.647128000	6.434967000	-4.770914000
C	-4.471495000	2.200238000	-5.041596000
C	-4.247544000	1.959766000	-0.666348000
C	-5.078061000	6.205177000	-0.391389000
O	-8.454255000	8.247123000	-4.122556000
C	-5.762120000	6.784214000	-6.239601000
C	-3.982579000	0.857750000	-5.326400000
C	-4.242757000	1.651284000	0.757478000
C	-5.351860000	7.622554000	-0.196750000
O	-7.644097000	10.214735000	-4.900952000
C	-5.786546000	5.391821000	-6.905417000
C	-3.743266000	0.259345000	-4.098519000
C	-4.509079000	2.837438000	1.414107000
C	-5.653369000	8.102934000	-1.475898000
C	-5.282352000	4.475324000	-5.791828000
C	-4.078694000	1.250889000	-3.084418000
C	-4.642391000	3.857610000	0.375782000
C	-5.511265000	7.003453000	-2.370470000
N	-4.516118000	3.282463000	-0.868261000
Mg	-5.227530000	4.061416000	-2.687135000
H	-3.641033000	7.021656000	-6.537374000
H	-4.446386000	8.503517000	-6.029872000
H	-2.349067000	-1.044879000	-3.074168000
H	-3.945633000	3.673515000	3.317391000
H	-4.621943000	2.059914000	3.380808000
H	-4.473229000	-2.305786000	-4.915839000
H	-3.144545000	-3.167290000	-3.941500000
H	-6.864736000	3.306015000	2.486646000
H	-6.076972000	4.727749000	3.227599000
H	-6.454153000	3.313990000	4.209018000
H	-5.444574000	9.438867000	-4.342644000
H	-9.234691000	10.000715000	-6.231307000
H	-8.977721000	11.635777000	-5.503222000
H	-9.708637000	10.302232000	-4.544132000
H	-4.862246000	2.825106000	-7.044997000
H	-3.712383000	0.040900000	-1.392921000
H	-4.830922000	5.565317000	1.630833000
H	-7.581870000	5.673471000	-8.119033000
H	-7.876253000	4.984060000	-6.503684000
H	-7.165620000	3.964338000	-7.784169000
H	-3.246953000	-0.695237000	-6.589897000

H	-2.966642000	0.944858000	-7.222555000
H	-4.585377000	0.230895000	-7.315392000
H	-4.553284000	-0.486424000	0.845558000
H	-4.208665000	0.271288000	2.411781000
H	-2.907292000	0.045441000	1.243720000
H	-5.949024000	9.250742000	1.072445000
H	-4.279839000	8.728888000	1.302026000
H	-5.579723000	7.722747000	1.913884000
H	-6.690109000	7.344443000	-6.445380000
H	-5.101600000	5.374212000	-7.766450000
H	-4.626722000	7.947537000	-7.713862000
H	-11.490202000	3.698130000	-2.935107000

3. Chl_{D1}

C	-10.810443000	-6.084505000	-7.523658000
C	-4.656624000	-12.511456000	-6.162534000
C	-2.086851000	-6.322250000	-1.919862000
C	-9.453513000	-3.174805000	-3.548861000
N	-8.959229000	-7.651019000	-5.258760000
C	-4.674525000	-13.639000000	-6.903597000
C	-1.784117000	-7.195385000	-0.692121000
C	-10.358640000	-4.171354000	-4.381471000
N	-6.705894000	-9.575810000	-5.245589000
O	-9.872844000	-2.086488000	-3.172643000
C	-11.527277000	-4.584377000	-3.515704000
N	-7.117667000	-5.803083000	-3.848199000
C	-13.878907000	-4.906592000	-3.428779000
C	-9.470323000	-5.381844000	-4.627178000
C	-8.969117000	-9.924217000	-6.150975000
C	-4.313763000	-9.804594000	-4.749128000
C	-4.964088000	-5.400834000	-2.794230000
C	-11.974033000	-8.964765000	-5.210545000
C	-7.760084000	-12.647446000	-7.092363000
C	-1.548951000	-9.020877000	-3.565779000
C	-6.300804000	-2.550577000	-2.097796000
C	-9.788603000	-6.564711000	-5.265661000
C	-7.638839000	-10.330944000	-5.900839000
C	-4.071383000	-8.588707000	-4.092731000
C	-6.256777000	-4.979111000	-3.127942000
O	-11.408013000	-5.018274000	-2.381801000
C	-10.985728000	-6.810167000	-6.165807000
C	-7.049995000	-11.573146000	-6.346732000
C	-2.800300000	-8.212284000	-3.502791000
C	-6.899212000	-3.715977000	-2.808069000
O	-12.707684000	-4.452534000	-4.150057000
C	-11.005647000	-8.354400000	-6.233663000
C	-5.710382000	-11.542247000	-5.963820000
C	-3.015336000	-7.014323000	-2.859525000
C	-8.184457000	-3.832327000	-3.356419000
C	-9.559060000	-8.696746000	-5.885178000
C	-5.520788000	-10.271669000	-5.272786000
C	-4.387033000	-6.628189000	-3.150955000
C	-8.248396000	-5.114500000	-3.978881000
N	-5.014366000	-7.622715000	-3.873103000
Mg	-7.088999000	-7.844699000	-4.151969000

H	-10.851956000	-4.997637000	-7.357655000
H	-11.647910000	-6.331614000	-8.193115000
H	-3.725839000	-12.295603000	-5.628967000
H	-2.511774000	-5.364438000	-1.598022000
H	-1.161982000	-6.058677000	-2.444190000
H	-5.544262000	-13.958276000	-7.472008000
H	-3.796184000	-14.283376000	-6.943107000
H	-1.020703000	-6.732963000	-0.056581000
H	-1.412671000	-8.184536000	-0.982845000
H	-2.690464000	-7.348193000	-0.093887000
H	-10.722467000	-3.664398000	-5.287865000
H	-14.679699000	-4.201527000	-3.661185000
H	-13.683742000	-4.920643000	-2.353062000
H	-14.132024000	-5.912539000	-3.787760000
H	-9.605646000	-10.659797000	-6.641167000
H	-3.453379000	-10.469661000	-4.833147000
H	-4.350703000	-4.717995000	-2.206916000
H	-11.937117000	-10.062699000	-5.234122000
H	-13.007100000	-8.643927000	-5.412732000
H	-11.708532000	-8.647165000	-4.191936000
H	-8.848941000	-12.544246000	-7.013838000
H	-7.481301000	-13.637873000	-6.708431000
H	-7.502316000	-12.640261000	-8.164814000
H	-1.678385000	-9.985375000	-3.055882000
H	-0.708712000	-8.510230000	-3.082171000
H	-1.257318000	-9.238113000	-4.605067000
H	-5.829213000	-1.863354000	-2.818794000
H	-5.532068000	-2.860943000	-1.379226000
H	-7.065265000	-1.969544000	-1.564867000
H	-11.913759000	-6.427356000	-5.716163000
H	-11.276821000	-8.705484000	-7.240718000
O	-8.124328000	-8.400456000	-2.453477000
H	-8.595619000	-7.657014000	-1.962026000
H	-7.684303000	-8.903141000	-1.731645000
O	-9.268408000	-6.408855000	-1.095814000
H	-9.972141000	-5.841189000	-1.478310000
H	-9.361138000	-6.352022000	-0.121204000
H	-9.879590000	-6.291951000	-8.051433000

4. Chl_{D2}

C	6.415649000	7.974332000	-8.721952000
C	0.709841000	15.156722000	-7.053634000
C	-2.364416000	9.044716000	-3.025333000
C	4.975949000	5.601465000	-4.392073000
N	4.531634000	9.842713000	-6.614432000
C	0.708470000	16.264213000	-7.819837000
C	-2.517624000	9.688443000	-1.637451000
C	5.878677000	6.474945000	-5.364676000
N	2.461400000	11.956535000	-6.462500000
O	5.393793000	4.572361000	-3.877829000
C	7.061247000	6.985115000	-4.567327000
N	2.684596000	8.234903000	-4.926269000
C	9.410053000	7.040598000	-4.317648000
C	4.993508000	7.647371000	-5.749177000
C	4.627599000	12.055273000	-7.637674000

C	0.202784000	12.477061000	-5.655440000
C	0.543865000	8.004084000	-3.779553000
C	7.612009000	11.086544000	-6.829793000
C	3.626527000	14.829991000	-8.533922000
C	-2.605898000	11.898438000	-4.389429000
C	1.820419000	5.232033000	-2.806917000
C	5.325354000	8.732010000	-6.535908000
C	3.377607000	12.590881000	-7.257290000
C	-0.136212000	11.271180000	-5.015737000
C	1.824087000	7.519203000	-4.094217000
O	6.955101000	7.689742000	-3.574372000
C	6.534605000	8.853739000	-7.451768000
C	2.883298000	13.892368000	-7.649385000
C	-1.424669000	10.986313000	-4.407385000
C	2.444340000	6.282615000	-3.658838000
O	8.247323000	6.597093000	-5.067641000
C	6.572574000	10.380195000	-7.710127000
C	1.632891000	14.041235000	-7.057488000
C	-1.324432000	9.728156000	-3.855443000
C	3.717139000	6.299122000	-4.257117000
C	5.155848000	10.806072000	-7.334855000
C	1.381978000	12.798589000	-6.329796000
C	0.016555000	9.246123000	-4.162820000
C	3.790893000	7.497314000	-5.025398000
N	0.710641000	10.209339000	-4.869171000
Mg	2.762272000	10.237263000	-5.358957000
H	6.467356000	6.910967000	-8.445783000
H	7.268890000	8.175040000	-9.387105000
H	-0.083945000	15.090306000	-6.303187000
H	-2.132541000	7.975097000	-2.920435000
H	-3.330448000	9.087694000	-3.552300000
H	1.441067000	16.447334000	-8.602465000
H	-0.046176000	17.032721000	-7.661465000
H	-2.780697000	10.751146000	-1.721058000
H	-1.586842000	9.618529000	-1.061404000
H	-3.304530000	9.195086000	-1.056281000
H	6.228025000	5.842588000	-6.194140000
H	9.265015000	6.863504000	-3.246999000
H	9.576502000	8.112525000	-4.490025000
H	10.243888000	6.436491000	-4.683844000
H	5.269342000	12.706428000	-8.233190000
H	-0.571776000	13.244845000	-5.640420000
H	-0.094176000	7.366678000	-3.166096000
H	7.549516000	12.178690000	-6.930059000
H	8.629885000	10.771263000	-7.102058000
H	7.452672000	10.836735000	-5.771035000
H	3.364155000	14.666438000	-9.590439000
H	4.711338000	14.683686000	-8.454353000
H	3.407329000	15.876366000	-8.294328000
H	-2.432628000	12.773722000	-3.742866000
H	-3.497990000	11.387377000	-4.004671000
H	-2.847053000	12.266772000	-5.398877000
H	1.685918000	4.292086000	-3.362744000
H	0.829027000	5.534548000	-2.450886000
H	2.446931000	4.984573000	-1.937269000
H	7.451887000	8.532292000	-6.933375000

H	6.787282000	10.607219000	-8.765825000
O	3.920034000	10.804230000	-3.762742000
H	4.208042000	11.696258000	-3.516107000
H	4.296514000	10.147331000	-3.079707000
O	4.847236000	8.982889000	-2.173282000
H	5.022543000	9.043072000	-1.209283000
H	5.577982000	8.459424000	-2.572351000
H	5.500383000	8.122249000	-9.295080000

5. Pheo$_{D1}$

C	-5.779036000	-14.313517000	-15.277842000
C	-4.907337000	-7.492629000	-9.022869000
C	0.209299000	-5.409055000	-14.730193000
C	-2.797078000	-12.001538000	-18.486612000
N	-5.257916000	-11.008703000	-14.473016000
C	-5.662823000	-7.415200000	-7.911420000
C	1.510787000	-6.227966000	-14.681531000
C	-4.001125000	-12.672822000	-17.693400000
N	-4.895731000	-8.985857000	-12.363174000
O	-2.303641000	-12.472652000	-19.505200000
C	-5.181422000	-12.911639000	-18.601436000
N	-2.862809000	-9.695975000	-15.846541000
C	-7.010419000	-11.912076000	-19.719031000
C	-4.235829000	-11.756494000	-16.502132000
C	-6.562341000	-10.793831000	-12.430886000
C	-3.507586000	-7.050514000	-11.751140000
C	-1.243064000	-7.884719000	-15.961042000
C	-8.556736000	-11.848560000	-14.740246000
C	-7.018706000	-9.869842000	-9.464056000
C	-1.395306000	-4.813619000	-11.980809000
C	-0.453504000	-9.411003000	-18.684038000
C	-5.222261000	-11.854905000	-15.536110000
C	-5.885278000	-9.749585000	-11.793682000
C	-2.673581000	-6.920389000	-12.879798000
C	-1.817318000	-9.041075000	-16.503887000
O	-5.459552000	-13.990434000	-19.106967000
C	-6.328609000	-12.895949000	-15.505877000
C	-6.099149000	-9.257547000	-10.457391000
C	-1.637838000	-5.887389000	-12.984425000
C	-1.513426000	-9.759819000	-17.697598000
O	-5.884880000	-11.787863000	-18.822222000
C	-7.191881000	-12.398905000	-14.319990000
C	-5.222133000	-8.192384000	-10.253510000
C	-0.977232000	-6.123641000	-14.163097000
C	-2.424971000	-10.831924000	-17.728571000
C	-6.305413000	-11.334271000	-13.691176000
C	-4.475852000	-8.006660000	-11.490155000
C	-1.634470000	-7.285642000	-14.762050000
C	-3.253940000	-10.754169000	-16.574788000
N	-2.663443000	-7.745453000	-13.961062000
H	-4.558754000	-9.106430000	-13.315700000
H	-3.205736000	-9.324859000	-14.948768000
H	-5.344063000	-14.331868000	-14.269382000
H	-4.959694000	-14.541861000	-15.975242000
H	-3.909515000	-7.044643000	-8.992562000

H	0.363024000	-4.473612000	-14.171267000
H	0.001972000	-5.111231000	-15.769769000
H	-6.666932000	-7.829200000	-7.844311000
H	-5.277216000	-6.920258000	-7.019610000
H	2.369031000	-5.618159000	-14.992000000
H	1.463381000	-7.111769000	-15.332849000
H	1.706068000	-6.578257000	-13.660074000
H	-3.644507000	-13.669761000	-17.393132000
H	-7.825242000	-12.449491000	-19.223171000
H	-6.717990000	-12.441020000	-20.634310000
H	-7.312983000	-10.885683000	-19.947701000
H	-7.374262000	-11.247514000	-11.863067000
H	-3.332766000	-6.336596000	-10.946110000
H	-0.410379000	-7.441405000	-16.504181000
H	-8.444292000	-11.009864000	-15.443057000
H	-9.118050000	-11.495769000	-13.866922000
H	-9.157462000	-12.629503000	-15.228328000
H	-7.911102000	-9.250669000	-9.291199000
H	-6.510069000	-9.962848000	-8.494975000
H	-7.355088000	-10.866362000	-9.775898000
H	-0.614426000	-4.126694000	-12.325943000
H	-1.065081000	-5.213135000	-11.011004000
H	-2.310000000	-4.227961000	-11.803491000
H	0.448447000	-9.042051000	-18.177002000
H	-0.788718000	-8.614154000	-19.364654000
H	-0.192257000	-10.284212000	-19.294948000
H	-6.904628000	-12.849476000	-16.446719000
H	-7.351812000	-13.208718000	-13.593782000
H	-6.531968000	-15.094958000	-15.380363000

6. Pheo$_{D2}$

C	1.286223000	15.625657000	-17.037650000
C	0.859739000	9.331298000	-10.315170000
C	-4.929460000	7.256077000	-15.423025000
C	-1.493314000	12.839529000	-20.223511000
N	0.760150000	12.554059000	-15.966991000
C	1.484424000	9.596059000	-9.151202000
C	-6.318892000	7.896633000	-15.582210000
C	-0.201586000	13.491147000	-19.554555000
N	0.331210000	10.918080000	-13.581624000
O	-1.868912000	13.100064000	-21.362053000
C	1.036964000	13.218693000	-20.386649000
N	-1.871157000	11.358117000	-17.058722000
C	2.896754000	11.778889000	-20.662797000
C	-0.181745000	12.946588000	-18.138267000
C	2.198267000	12.460921000	-14.007331000
C	-1.149931000	9.168329000	-12.689701000
C	-3.650400000	9.721273000	-16.826775000
C	4.009645000	12.927778000	-16.622598000
C	3.014077000	11.582731000	-11.117030000
C	-3.199944000	6.868045000	-12.714293000
C	-4.210751000	10.652380000	-19.884495000
C	0.770151000	13.194240000	-17.165694000
C	1.483125000	11.564049000	-13.209506000
C	-2.113536000	9.038890000	-13.714063000

C	-2.978344000	10.691322000	-17.587368000
O	1.446524000	13.923493000	-21.291498000
C	1.882655000	14.220284000	-17.267745000
C	1.841624000	11.097352000	-11.895673000
C	-3.118824000	7.965582000	-13.719788000
C	-3.143991000	11.105422000	-18.944743000
O	1.649792000	12.064006000	-20.008648000
C	2.816954000	13.755969000	-16.126133000
C	0.910616000	10.128591000	-11.525196000
C	-3.853234000	8.134345000	-14.865712000
C	-2.070763000	11.984315000	-19.206409000
C	1.877033000	12.894939000	-15.292618000
C	-0.055531000	10.017763000	-12.614347000
C	-3.270367000	9.288980000	-15.551978000
C	-1.294041000	12.100756000	-18.019282000
N	-2.227498000	9.824290000	-14.818271000
H	-0.116971000	11.044840000	-14.487128000
H	-1.572067000	11.153798000	-16.093642000
H	0.945818000	15.682767000	-15.992874000
H	0.389989000	15.773469000	-17.659902000
H	0.208087000	8.454367000	-10.345159000
H	-5.018959000	6.364726000	-14.785007000
H	-4.600206000	6.886424000	-16.407143000
H	2.097849000	10.478280000	-8.984419000
H	1.340113000	8.936606000	-8.296122000
H	-6.943606000	7.278408000	-16.241013000
H	-6.259590000	8.899479000	-16.026671000
H	-6.846652000	7.980818000	-14.624487000
H	-0.342427000	14.579811000	-19.583054000
H	2.742491000	11.527151000	-21.721742000
H	3.315884000	10.920461000	-20.127049000
H	3.576343000	12.639280000	-20.594941000
H	3.118546000	12.854169000	-13.574910000
H	-1.273969000	8.497527000	-11.839668000
H	-4.486802000	9.211970000	-17.304421000
H	4.622369000	12.568549000	-15.784156000
H	4.650324000	13.528568000	-17.283192000
H	3.661613000	12.050460000	-17.189029000
H	3.733955000	12.120120000	-11.747047000
H	3.533587000	10.743825000	-10.636349000
H	2.704844000	12.266079000	-10.310868000
H	-2.288221000	6.251237000	-12.746649000
H	-4.061322000	6.214462000	-12.903874000
H	-3.285528000	7.248213000	-11.688114000
H	-5.086310000	10.270547000	-19.341919000
H	-3.844674000	9.846431000	-20.541702000
H	-4.533665000	11.477588000	-20.535018000
H	2.391165000	14.164777000	-18.244859000
H	3.188517000	14.603300000	-15.530820000
H	1.977155000	16.441291000	-17.250773000

PBE/Def2-TZVP Gas-Phase Optimized Geometry of Chlorophyll *a*

C	-6.96955532980476	35.83164086066760	8.03087168346602
C	-3.97831446364572	34.71442905049682	-0.71308596099324
C	2.45801151962580	37.09786154429551	3.41015181100734
C	-2.25026671626436	35.85967478018361	9.96992474855971
N	-4.58659058760425	34.88156724656598	5.84430804701611
H	-6.98958954258222	36.57850224351265	7.22343249572018
C	-4.89233224915220	34.72948689924828	-1.70223098938268
C	2.52385946996387	38.62831126608160	3.53936219159616
C	-3.74599807311062	35.42120662514685	9.57471435664959
N	-3.77223904702334	34.97699286375801	2.97409194529472
O	-1.87383718468599	36.02293371624963	11.11651349952769
C	-4.09203904321669	34.12740795346006	10.27977253109801
N	-1.85831276169031	35.81191110379756	6.44112915379949
C	-4.82281511907293	33.21105520941798	12.32358878357142
C	-3.73610606053716	35.33027716483542	8.05963353418130
C	-5.92528970775784	34.22783557201340	3.89514612776429
C	-1.92803971175853	35.53689316425194	1.44864988062107
C	0.36022880270147	36.45692962956236	5.65072406411389
C	-6.79236886599198	32.41253494646393	6.39593124954742
C	-6.64031937301997	33.80542421905502	0.87353634555162
C	0.94793893748929	36.46968974618806	0.63596432033778
C	0.94177079268199	36.75803224725851	8.78956480066019
C	-4.75622380891666	34.94904232325437	7.20743091331833
C	-5.05202006781342	34.50967153667435	2.82490358112479
C	-0.92991324334456	35.90740357907517	2.36906362609743
C	-0.54813174111192	36.22159822221447	6.68973612285199
O	-3.93119799304285	33.00906596236503	9.82885145666293
C	-6.17460823102262	34.59020996249630	7.60195831178209
C	-5.35849264033969	34.33542229852746	1.42029928321632
C	0.41389822242334	36.33553271306462	2.02406126481293
C	-0.32594555069147	36.35236474374192	8.11836227341463
O	-4.58073709434601	34.38187257126852	11.51799599260500
C	-6.72225844141453	33.94328099918019	6.30815144852252
C	-4.22033174139175	34.71372501976756	0.71606414645017
C	1.06386186242389	36.58964979459695	3.21533443017390
C	-1.54635156551351	36.00789345544521	8.70421046205845
C	-5.71068747576708	34.38347673631521	5.26017815683008
C	-3.22780166128736	35.10277567822087	1.71318573719710
C	0.10805657613462	36.32086507410688	4.27691472404557
C	-2.43920267987864	35.68994099652298	7.64205750443535
N	-1.09128831387975	35.90893933672996	3.73011409984025
Mg	-2.82908677152284	35.39254551907499	4.72337394089593
H	-6.52924865915722	36.30486166012889	8.91909448387902
H	-8.00731053865120	35.55832942094849	8.27203244496026
H	-2.92414538058384	34.70714595736468	-1.00899440381850
H	3.08680002802214	36.77746790380147	2.56459371405324
H	2.90197812048313	36.63588674622336	4.30667032059589
H	-5.96379662293370	34.78007986612220	-1.51332031097650
H	-4.57814101692053	34.71912524256556	-2.74608102450737
H	3.55867848830001	38.96942775066955	3.68827003164468
H	1.92097918095002	38.97661643942445	4.39010883661435
H	2.12915290783496	39.11245008606240	2.63474380195741
H	-4.40195652559290	36.21463927293521	9.96605403960215
H	-5.19709578144030	33.58757420563515	13.28076507620918

H	-3.89182642716462	32.64683241500218	12.46618879623649
H	-5.56498856074647	32.55882189634930	11.84372442131859
H	-6.90395466887648	33.83114597727975	3.62011159735627
H	-1.64454379926567	35.59646372317199	0.39704499642482
H	1.36294784211267	36.78092893583597	5.93526832059795
H	-7.10269209982149	31.97218692157744	5.43796722506948
H	-7.51302112794546	32.10349047217936	7.16722942589882
H	-5.80946051293792	31.99653072793389	6.66114446142932
H	-6.45610328543132	33.16376714798754	-0.00100706944107
H	-7.31295218976082	34.61434054515752	0.54286687581539
H	-7.18576188492248	33.21368464240687	1.62087530442276
H	2.01964951049906	36.70858030111329	0.64196068664663
H	0.43520479229202	37.27068036564848	0.07955188329943
H	0.81647434269838	35.54170567440227	0.05803091212039
H	0.79472655720226	36.84120984694055	9.87300989840401
H	1.30604949455989	37.72643943992408	8.41269141892819
H	1.74220520217878	36.02263437339533	8.60959099626279
H	-6.16636801731903	33.86158602379247	8.42730063398050
H	-7.72274823890209	34.33636840986109	6.06599257797070
